# Radiation protection following nuclear power accidents: a survey of putative mechanisms involved in the radioprotective actions of taurine during and after radiation exposure

**DOI:** 10.3402/mehd.v23i0.14787

**Published:** 2012-02-01

**Authors:** Olav Albert Christophersen

**Keywords:** ionizing radiation, radiation illness, therapy, rheumatoid arthritis, asthma, diarrhoea

## Abstract

There are several animal experiments showing that high doses of ionizing radiation lead to strongly enhanced leakage of taurine from damaged cells into the extracellular fluid, followed by enhanced urinary excretion. This radiation-induced taurine depletion can itself have various harmful effects (as will also be the case when taurine depletion is due to other causes, such as alcohol abuse or cancer therapy with cytotoxic drugs), but taurine supplementation has been shown to have radioprotective effects apparently going beyond what might be expected just as a consequence of correcting the harmful consequences of taurine deficiency *per se*. The mechanisms accounting for the radioprotective effects of taurine are, however, very incompletely understood. In this article an attempt is made to survey various mechanisms that potentially might be involved as parts of the explanation for the overall beneficial effect of high levels of taurine that has been found in experiments with animals or isolated cells exposed to high doses of ionizing radiation. It is proposed that taurine may have radioprotective effects by a combination of several mechanisms: (1) during the exposure to ionizing radiation by functioning as an antioxidant, but perhaps more because it counteracts the prooxidant catalytic effect of iron rather than functioning as an important scavenger of harmful molecules itself, (2) after the ionizing radiation exposure by helping to reduce the intensity of the post-traumatic inflammatory response, and thus reducing the extent of tissue damage that develops because of severe inflammation rather than as a direct effect of the ionizing radiation *per se*, (3) by functioning as a growth factor helping to enhance the growth rate of leukocytes and leukocyte progenitor cells and perhaps also of other rapidly proliferating cell types, such as enterocyte progenitor cells, which may be important for immunological recovery and perhaps also for rapid repair of various damaged tissues, especially in the intestines, and (4) by functioning as an antifibrogenic agent. A detailed discussion is given of possible mechanisms involved both in the antioxidant effects of taurine, in its anti-inflammatory effects and in its role as a growth factor for leukocytes and nerve cells, which might be closely related to its role as an osmolyte important for cellular volume regulation because of the close connection between cell volume regulation and the regulation of protein synthesis as well as cellular protein degradation. While taurine supplementation alone would be expected to exert a therapeutic effect far better than negligible in patients that have been exposed to high doses of ionizing radiation, it may on theoretical grounds be expected that much better results may be obtained by using taurine as part of a multifactorial treatment strategy, where it may interact synergistically with several other nutrients, hormones or other drugs for optimizing antioxidant protection and minimizing harmful posttraumatic inflammatory reactions, while using other nutrients to optimize DNA and tissue repair processes, and using a combination of good diet, immunostimulatory hormones and perhaps other nontoxic immunostimulants (such as *beta*-glucans) for optimizing the recovery of antiviral and antibacterial immune functions. Similar multifactorial treatment strategies may presumably be helpful in several other disease situations (including severe infectious diseases and severe asthma) as well as for treatment of acute intoxications or acute injuries (both mechanical ones and severe burns) where severely enhanced oxidative and/or nitrative stress and/or too much secretion of vasodilatory neuropeptides from C-fibres are important parts of the pathogenetic mechanisms that may lead to the death of the patient. Some case histories (with discussion of some of those mechanisms that may have been responsible for the observed therapeutic outcome) are given for illustration of the likely validity of these concepts and their relevance both for treatment of severe infections and non-infectious inflammatory diseases such as asthma and rheumatoid arthritis.

## Introduction

The strength 9 (Richter scale) earthquake and attending tsunami that recently took place in Japan have led to severe damage to several nuclear reactors. This article has been written with the purpose that it hopefully might be helpful both for those physicians who now have the direct responsibility to take care of patients who have been exposed to high levels of ionizing radiation (while working with the damaged nuclear power plants in an effort to hinder the release of much larger quantities of radionuclides from the reactors), for the health authorities in Japan and for others who participate in international efforts to assist Japan with minimizing the health impact of this severe disaster. Because this survey report has been written in an emergency situation in the hope that the information presented might be useful for Japanese physicians and their patients before it is too late, there has not been enough time available to study all relevant literature in the way an author of a scientific survey article will normally try to do. This means that there very likely will be weak points in this text that might have been discovered if more time had been available for the author – and also more time for criticism of the manuscript by colleagues and critical discussions with them.

However, it is hoped that the author partly might compensate for this weakness by drawing on his experience from several years’ work with partly related (overlapping or parallel) problems in connection with other severe diseases (such as HIV disease and hypervirulent influenza), as well as on his personal experience through several years not only as an allergy patient but also as a patient suffering from chronic poisoning with a substance most likely functioning as a potent reactive oxygen species (ROS)-generating redox cycling agent (like some kind of lipid-soluble analog of the herbicide paraquat), and from successful antidote therapy of this potentially very serious poisoning and associated asthma. Knowing that this survey report is far from perfect, critique from readers will be welcome. However, it is hoped that rather than leading to too much theoretical discussion about moot points (which in the absence of new experimental observations might be more or less futile), this paper may instead function as a stimulus to carry out new experimental studies either in the lab or in form of clinical studies for every important question that cannot yet be settled by observations already available. It is hoped that it may serve not only as a survey of now very well-documented research observations (which, however, are not always utilised practically in clinical medicine as might have been expected, even when from a purely scientific point of view there can not be any reasonable doubt about the validity of the observations), but also as an eye-opener to important questions still imperfectly understood.

## The effects of ionizing radiation on taurine excretion and of taurine as a radioprotectant are well documented by animal experiments

There is a good deal of experimental literature (with some of it coming from Japan) about protective effects of taurine and taurine derivatives against biological damage caused by ionizing radiation, but much of this literature is now very old, perhaps so old that it may have been forgotten, and not easily available (if at all) by searching for it on PubMed. Some of the articles are so old that abstracts are not given on PubMed, which means one cannot know what was the outcome of the experiment without studying the original. Sometimes they may also have been published in journals or books not included in this literature data base. As examples of literature demonstrating radiation protection by taurine or taurine derivatives in cultured cells or animals, see references 1–9.

Several studies have shown that taurine excretion is enhanced following exposure to high doses of ionizing radiation (10–21), and taurine depletion has also been observed in the blood plasma of human cancer patients after cytotoxic chemotherapy and/or radiation therapy without any change in the blood plasma concentrations of the taurine precursor amino acids methionine and cysteine (22). The enhanced urinary excretion of taurine following exposure to high doses of ionizing radiation can most plausibly be explained as a consequence of enhanced passive leakage of taurine out of cells that have been exposed to much ionizing radiation because of damage to their plasma membrane. Something similar may presumably happen during cancer therapy with cytotoxic drugs that often function as potent oxidant stressors, and therefore may cause taurine release not only from those tumor cells which they kill, but from normal host cells as well as a result of damage to their plasma membranes. This article may hopefully help to make it clear why it may be very important for the quality of life and perhaps also survival possibilities of cancer patients that taurine depletion happening as a consequence of rough therapy should be corrected.

While taurine administration before or during irradiation conceivably might have protective effects because taurine functions as an antioxidant protectant, taurine administration afterwards might be beneficial by compensating for taurine losses that have occurred because of the irradiation, thus helping to correct harmful effects of taurine depletion *per se*. However, it will be shown below that taurine supplementation beyond normal physiological requirements may be beneficial because of the effects both of taurine itself and the taurine metabolites taurine chloramine and taurine bromamine as anti-inflammatory signal substances. Taurine may, moreover, also exert an important antifibrogenic effect.

## Evidence showing that taurine functions as a biological antioxidant

Evidence that taurine functions as an important biological antioxidant is in large measure indirect, because taurine has been shown to function as a good antidote against several different toxic substances that have little in common, when considering the whole group of substances concerned, other than acting as oxidant stressors, *i.e*. as prooxidants, either directly when the substance concerned is a reactive oxidant molecule, indirectly via reactive metabolites (that *e.g*. might function as ROS-generating redox cycling agents) or because they inhibit important antioxidative enzymes. Such antidote effects of taurine or high capacity for taurine uptake into the cells (or conversely harmful effects of taurine depletion) have *i.a*. been found against alcohol (23–40), against the analgesic and fever-depressing drug acetaminophen (also called paracetamol) (41–44), against the aminoglycoside antibacterial drug gentamicin (45–47), against the immunosuppressant drug cyclosporine A (48, 49), against the anticancer drugs cisplatin (50–54), doxorubicin (also called adriamycin) (55–62), bleomycin (63–78), methotrexate (79) and taumustine (80), against the cytokine interleukin-2 (IL-2), which is used for immunotherapy of cancer (81–84), against the estrogen receptor modulator tamoxifen, which is used in therapy of breast cancer (85–87), against the antiarrhythmic drug amiodarone (88), against the *beta*-adrenergic agonists isoproterenol (55) and isoprenaline (89–92), against nicotine (93–95), against oxidized low-density lipoprotein (LDL) (96), against oxidized fish oil (97), against high glucose levels (96, 98–110), against fructose (111–122), against galactose (123), against advanced glycation end-products (AGEs) (100, 124, 125), against homocysteine (126–130), against the anti-inflammatory drugs ibuprofen (131) and indomethacin (132, 133), against the herbicide paraquat (65, 134, 135), against carbon tetrachloride (136–149), against menadione (150), against 1,4-naphtoquinone (137, 139), against hydrazine (137, 139), against trinitrobenzene sulfonic acid (151, 152), against acrylonitrile (153), against perchloroethylene (154), against methylene dianiline (155), against arsenic (156–164), against cadmium (165–172), against mercury (173), against lead (174–179), against copper (180), against iron (39, 181–183), against ozone (184–188) and against NO_2_ (189). The studies on antidote effects of taurine in animals poisoned by bleomycin and amiodarone are of special interest because they show a very important antifibrogenic effect of taurine in the lower respiratory tract, which may be further enhanced by combining taurine with high doses of niacin. This might be very relevant both for treatment of patients with chronic obstructive pulmonary disease (COPD) – in an attempt to delay the progression of this disease – and for prophylaxis of fibrosis in patients who have been exposed to high levels of ionizing radiation.

Taurine has, moreover, also been reported to function as a good scavenger of various aldehydes (when the amino group of the taurine molecule reacts with aldehyde groups) (190–192), which might be relevant when large quantities of acetaldehyde are formed following ingestion of too much alcohol, but also in diabetes. It also reacts rapidly with hypohalite ions, such as hypochlorite and hypobromite (193–197), which are formed during inflammatory reactions by haloperoxidases such as myeloperoxidase and eosinophil peroxidase when the respiratory burst of leukocytes such as neutrophils, eosinophils or monocytes is activated. Since there is very abundant literature, which easily can be found by searching on PubMed (simply by using taurine chloramine and taurine bromamine as search words), about the reactions between taurine and hypohalite ions and about the effects of the reaction products, only some few examples are given here. It should be noted that while the reaction products taurine chloramine and taurine bromamine are less reactive than their parent compounds hypochlorite and hypobromite, they are still reactive molecules with oxidant properties; they are absolutely not antioxidants. However, they have anti-inflammatory effects following uptake in human cells (198–201) which happens partly because these substances and also glycine chloramine inhibit activation of the transcription factor NF-*kappa*B (202–205), which is a positive regulator of several different genes with proinflammatory effect (206–208).

There are, additionally, also several studies showing that taurine protects (in the same organ or other parts of the body) against damage caused by ischemia and reperfusion in several different organs: in the heart and isolated cardiomyocytes (209–230), in skeletal muscle or whole limbs (231–235), in the liver (236–247), in the kidneys (248–250), in the testicles (251–253), in the intestines (254), in peripheral nerves (255), in the lungs (256) and in the brain (257–265). There are, however, also some reports showing no protective effect of taurine supplementation, or a paradoxical protective effect of poor taurine status, so all literature data are not consistent. However, the reports where a protective effect of taurine was found are far more numerous than those showing no effect or an apparent protective effect of poor taurine status.

It is likely that this protective effect of taurine in ischemia followed by reperfusion may be due to more than one mechanism, and that regulatory effects of intracellular taurine on membrane transport systems for calcium may be part of the reason why taurine is protective in tissues that have been exposed to ischemia followed by reperfusion. But since it is well documented that ischemia followed by reperfusion is attended by strongly enhanced oxidative stress, with only some few examples of the very abundant literature about this being given here for illustration (266–275), there must be good reason to suspect that the protective effect of taurine against organ damage caused by ischemia and reperfusion may in large measure be explained by its antioxidant effect in living cells. This is supported by abundant experimental data showing that other substances with antioxidative protective effects also have similar protective effects against tissue damage caused by ischemia/reperfusion (not only in the affected organs themselves, but a protective effect was in some studies found also against remote injury in other organs), or that depletion leads to enhanced tissue injury, which has been abundantly demonstrated *e.g*. for selenium and the selenium-dependent antioxidant protective enzyme glutathione peroxidase (272, 276–311), for glutathione and glutathione precursors (312–337), for carnosine (338–347) and for melatonin (346, 348–474), but the same also has been reported for various other biological antioxidants. It can also be directly seen from many of the reported experimental studies concerning protective effects of taurine against ischemia/reperfusion damage that this protection must very likely be partly mediated by an antioxidative protective effect, as one can see reduction of the concentrations of direct and/or indirect oxidative stress indicators, compared with the control group, after taurine supplementation.

Since there is much overlap between those chemical mechanisms that cause cellular and organ damage during ischemia followed by reperfusion and those causing cellular damage during exposure to ionizing radiation, with free radicals and other energy-rich oxidant molecules (such as H_2_O_2_, peroxynitrite and singlet molecular oxygen) playing a central role not only during ischemia/reperfusion, but also when cells and organs are damaged by ionizing radiation (475), there is good reason to believe that most of the experimental data showing protective effects of antioxidant nutrients and the antioxidant hormone melatonin against damage caused by high levels of ionizing radiation also may be relevant for antiischemic protection, and vice versa. The same substances that can protect various organs against damage caused by ischemia and reperfusion may be expected, in a great majority of cases, to be protective against radiation injury as well (which has in several cases also been confirmed by experimental studies), and most substances that can protect against radiation injury would also be expected to have protective effects against tissue damage caused by ischemia and reperfusion.

There is, moreover, much overlap comparing biochemical pathogenetic mechanisms in ischemia/reperfusion and radiation sickness with the biochemical pathogenetic mechanisms in acute or chronic poisoning by substances functioning as strong oxidant stressors (with paraquat and the fungal poison orellanine as typical examples), which explains why several substances that have been found in animal experiments to protect against tissue damage caused by ischemia/reperfusion also have been found to function as good antidotes against several different toxic substances with a prooxidant mechanism of action. There is a very voluminous research literature about this, but most of it is not much practically utilised, not even when the protective substance concerned both is very non-toxic and cheap and presumably can be administrated in high doses with very little risk of serious side effects. Many examples of animal experiments demonstrating such antidote effects can easily be found by combining as search words on PubMed the names of various substances mentioned above for which taurine has been found to function as an antidote (*e.g*. gentamicin, ethanol or cadmium) with the names of other antioxidant nutrients (*e.g*. selenium, for which there is an abundant research literature, especially concerning the use of selenium as an antidote against toxic heavy metals and arsenic) or of endogenously produced antioxidants (*e.g*. melatonin and coenzyme Q_10_), as well as of polyphenolic plant antioxidants (*e.g*. silymarin). The present author is himself a chronic poisoning patient who would probably not have been alive without such antidote therapy (having for several years used a combination of high doses of selenium, high doses of coenzyme Q_10_, high doses of vitamin E and high doses of protein- and taurine-rich fish powder).

Strong parallels may also be found between all these disease situations and what happens during severe inflammatory conditions, both during severe infection (*e.g*. malignant avian influenza), non-infectious inflammatory diseases including rheumatoid arthritis and asthma, and following severe trauma, *e.g*. severe burn injuries or mechanical trauma to the brain. During infection, oxidative stress is enhanced partly as a consequence of the use of strong oxidants (such as hypohalite ions and peroxynitrite) as antibacterial and antiviral weapons, and partly because of cytokine-induced enhancement of mitochondrial ROS production. But the same mechanisms leading to enhanced oxidative stress will also be activated during various non-infectious inflammatory conditions, as during allergic inflammation, and also following severe trauma (*e.g*. severe burn injuries).

For obtaining an optimal therapeutic response, it is probably best in all these cases to use a cocktail of protective substances rather than one substance alone, since there are good theoretical reasons to believe that many of the substances concerned may interact with another in a synergistic fashion, especially when they have completely different biochemical functions or different localization in the cells (*e.g*. water-soluble versus lipid-soluble antioxidants), or otherwise have completely different mechanisms of action (*e.g*. free radical scavengers versus redox-active metal ion chelators).

It may also be important to use high enough doses of each substance and take into consideration their pharmacokinetic behaviour, especially for such substances that are rapidly excreted following intake of high doses (such as taurine because of limited capacity for tubular reabsorption in the kidneys) or have a rapid normal turnover because they are rapidly degraded by enzymes (such as melatonin, which must have a rapid turnover because of its role as a hormone participating in the regulation of diurnal rhythms) or by rapid non-enzymatic *in vivo* oxidation processes (as is probably the case for coenzyme Q_10_). It might thus be an advantage to use slow-release high-dose preparates both in the case of taurine and melatonin in order to obtain a more prolonged effect following intake of the pill (while for coenzyme Q_10_, turnover is slow enough that this should not be necessary). Such slow-release high-dose taurine and melatonin preparates are perhaps not commercially available today (certainly not in Norway, but it might perhaps also be difficult to find them in other countries), but they should be easy to make, even for use in acute situations like now following the nuclear accident in Japan.

### Should a combination of high-dose melatonin and antioxidant nutrients be used as part of the standard routine for acute therapy of brain stroke, myocardial infarction and similar disorders?

In this article, I have included far more literature references than normal even for a survey article. This is because I believe it may be useful to have too many rather than too few references for those in Japan who in the present emergency situation hopefully might find some of the information I have tried to survey to be useful in their work, when trying to limit as much as possible the health damage caused by the nuclear power plant disaster.

The strength of documentation for a certain biological effect of some given protective substance does not only depend on the quality of individual research reports, but also on their total number as well as on their diversity. When the same effect has been found in several different organs by several different groups of scientists, this is much more compelling evidence than when it has been found only in one organ by one group of scientists. And it is better when not only an overall protective effect of some particular intervention has been demonstrated in a particular organ, but also important parts of the responsible mechanism (or mechanisms) have been explained by experimental observations. One has therefore to be convinced about the validity of the observations, concerning for instance the protective effects against tissue damage caused by ischemia and reperfusion of substances such as taurine, selenium, glutathione and melatonin, by the sheer abundance of good quality research reports, as well as by the number of different organs where similar protective effects have been found, at the same time as much of the mechanisms explaining the protective effects appear to be reasonably well understood.

This is especially important in such cases where something of potential value in practical medicine has been well studied in animal experiments, but still not in human patients in well-conducted clinical trials, with such trials either being few or not existing at all. The laws of physics and chemistry are, nevertheless, the same, regardless of biological species from methanogens to man (and, as far as we can see, everywhere in the observable Universe). And most of the fundamental biochemistry, intracellular signal pathways and extracellular signal substances (including hormones and cytokines) are also the same in all mammalian species, which makes it possible to extrapolate with confidence from much of what has been observed in experiments with one species, *e.g*. rats, also to other mammalian species, including humans.

One should, of course, be cautious when carrying out such extrapolation and bear in mind those important differences that exist due to different ecological niche adaptations in different species. But with application of experience and good judgement, it is possible to understand the difference between such phenomena that might be different in different species and families of animals because they may be influenced by their different ecological niche adaptations (*e.g*. when comparing digestive physiology in ruminants and monogastric species, or the immune system in very short-lived mammalian species and long-lived ones) and such phenomena that are so fundamental and universal that they must be expected to be the same everywhere (as *e.g*. in the case of reactive oxygen species (ROS) reacting with DNA molecules, with ROS scavenger molecules having antimutagenic effect).

It is very strange, when considering how abundant those research data are that show very significant protective effect for some of the substances mentioned here against permanent organ damage caused by ischemia/reperfusion, and how non-toxic and cheap some of them (like taurine, melatonin and glutathione) also are, that the use of these substances has not already become standard routine, *e.g*. for treatment of stroke patients or patients with myocardial infarction, but also for treatment of other diseases and injuries where permanent organ damage develops as a consequence of ischemia/reperfusion and post-injury inflammatory response (*e.g*. severe head trauma and drowning). It can hardly be considered knowledge-based or research-based clinical medicine not to utilise data from basal research when they are of so good quality and also so abundant as is demonstrated by the very long literature list of this article, especially for the protective effect of melatonin against damage caused by ischemia and reperfusion in several different organs, but also for taurine.

It is very difficult to find any legitimate scientific reason why this should be so. Rather, the explanation must somehow be at the level of communication (between basal scientists and clinicians), sociology or economy, *e.g*. in the way clinical research is funded (with too small proportion of the funding of clinical research projects coming from the user and buyer side compared with the proportion of research money coming from the seller side, which means that far too little money is used for clinical research on such substances or methods which could help to make practical medicine not only better, but also much cheaper than it is today). The health economic savings for society if the abundant animal experimental data concerning protection against tissue injury caused by ischemia/reperfusion might be better utilised than now (with much better acute treatment for stroke and myocardial infarction than is common today, *e.g*. by combining a strong antiischemic protective cocktail with hypothermia) would be potentially enormous, not least for those countries (including Japan), that have a high relative proportion of elderly, when considering the age distribution of the whole population, and where the proportion of elderly can be expected to increase even more during the next decade.

### Use of a combination of slow-release, high-dose taurine and melatonin preparates in combination with high-dose coenzyme Q_10_ and other antioxidants for antidote therapy of acute or chronic poisoning with substances with a prooxidant mechanism of action

Slow-release high-dose taurine and melatonin preparates might probably be helpful in the future also for treatment of several other diseases and injuries, including acute intoxications with substances having a prooxidant mechanism of action (such as paraquat or the fungal poison orellanine) – in which case it is probably also much better to use a suitable antioxidative antidote cocktail rather than one substance alone.

For treatment of acute poisoning with substances functioning as oxidant stressors, it is presumably best to use a combination both of lipid-soluble and water-soluble antioxidants including melatonin (at a high dosage level) and taurine, as well as coenzyme Q_10_ for preventing too much disturbance of the energy metabolism of vulnerable target organs (like the kidneys in the case of orellanine poisoning) because of rapid peroxidative coenzyme Q_10_ degradation. It may also be an advantage for obtaining a rapid therapeutic response in acute situations (*e.g*. following orellanine ingestion) to rely on high doses of non-toxic substances having a rapid turnover in the human organism, rather than relying too much on such substances that have a much slower turnover and therefore need much more time to build up to new steady state intracellular concentrations.

## Possible mechanisms explaining the antioxidative protective effects of taurine

While the reactions between the amino group in the taurine molecule on one side and aldehyde group and hypohalite ions on the other are well documented and easy to understand, these reactions can not alone explain the multiple protective effects of taurine against several different substances with prooxidant action, *i.e*. against molecules which either themselves are reactive and function as oxidant stressors (such as NO_2_ and paraquat), which can be metabolized into molecules that function as oxidant stressors, *e.g*. because an inactive parent substances is converted by hydroxylation to form a ROS-generating redox cycling agent, or can inhibit antioxidative enzymes (which may be an important part of the mechanism of the toxic effect for a number of toxic metals). But reports conflict with another regarding the efficacy of taurine as a scavenger of molecules important as oxidants in living organisms, other than those already mentioned.

### Role of taurine as a scavenger of reactive oxidant molecules

Aruoma et al. reported in 1988 that taurine does not react rapidly with O_2_^–^, H_2_O_2_ or OH^–^, and the product of its reaction with HOCl is still sufficiently oxidizing to inactivate *alpha* 1-antiproteinase (476). They concluded on this background that it seems unlikely that taurine functions as an antioxidant *in vivo*. By contrast, they found hypotaurine to be a much better antioxidant than taurine (476). In a study by another group several years later, taurine's capacity to scavenge peroxynitrite was measured, and it was concluded that taurine is only a weak scavenger of peroxynitrite, and that it does not attenuate sodium nitroprusside toxicity to cells in culture (477).

In a more recent study (from 2010), Oliveira et al. found also that taurine does not react with H_2_O_2_ (478). However, taurine was found to exhibit significant scavenging potential against peroxyl radical, nitric oxide, and superoxide donors (478). Their study also evaluated if taurine was able to minimize the *in vitro* Cu/Zn-superoxide dismutase damage (SOD) induced by peroxynitrite (478). Taurine was found to prevent both the formation of nitrotyrosine adducts and the decrease in SOD activity caused by peroxynitrite (478). In addition, taurine was found to prevent the *ex vivo* damage caused by *tert*-butyl hydroperoxide in rat liver slices (478). Oliveira et al. concluded from these experimental data that taurine at different physiological concentrations efficiently scavenges many reactive oxygen and nitrogen species (478).

### Possible role of taurine as an inhibitor of oxidation reactions caused by perferryl ion complexes and iron-catalyzed reactions between organic molecules and H_2_O_2_

From these partly conflicting reports, one may conclude that taurine is not free of scavenger activity that partly might help to explain its *in vivo* antioxidant protective effects, but it is not strikingly effective as a scavenger antioxidant except for hypohalite ions and aldehydes. There are many other biological antioxidants that also scavenge reactive molecules, but are more effective than taurine – even though taurine might partly compensate for modest specific effect (at a given concentration) by much higher *in vivo* concentrations than for many other biological antioxidant molecules.

It should be remembered, however, that it is also possible for a chemical substance to function as a good antioxidant in non-living or living systems without having any scavenger effect at all either for free radicals or for other (non-radical) important oxidant molecules, such as H_2_O_2_, peroxynitrite and singlet molecular oxygen. This is because of the crucial role played by iron and other redox-active metals as catalysts of non-enzymatic oxidation reactions both in living organisms (479–493) and non-living systems (*e.g*. during storage of foods or animal feeds in contact with air, or in rubber). Substances that can form complexes with such catalytically active metals in a way making them inactive as catalysts will therefore also function as good antioxidants, and can be used for this purpose *e.g*. in order to enhance the shelf life of various food products.

Citrate is a good example of substances used for this purpose by the food industry. It inactivates the metal atom (hinders it from being catalytically active) by forming a chelate complex, thus hindering reaction between the metal atom and such oxidant molecules that are important during oxidative rancidification, such as molecular oxygen, H_2_O_2_, and organic hydroperoxides. At the same time, citrate is also a substance normally formed by human cells, where it is removed as rapidly as it is formed, and completely nontoxic at the levels in which it either is added or can be naturally found in foods.

Taurine, unlike citrate, does not have a structure making it a strong chelator for divalent or trivalent metal ions. But at the high concentrations at which it normally occurs inside living cells, it will instead be capable of forming mixed complexes with iron together with other organic molecules. A type of iron complex that seems to be especially important as a catalytic agent for undesirable spontaneous oxidation processes in living cells are those complexes that can form between iron and organic phosphate molecules, both small ones (such as AMP, ADP, ATP and phospholipids) and macromolecules, especially in form of nucleic acids (DNA and RNA). The reaction between H_2_O_2_ and DNA molecules is very sluggish in the absence of redox-active metal atoms that can catalyze the reaction. But this reaction is readily catalyzed both by iron and other redox-active metal ions (including uranium and plutonium, which perhaps might bind even more strongly to DNA compared to iron atoms and hence might be correspondingly more poisonous as catalysts of the reaction between DNA and H_2_O_2_), for which reason substances that can bind these metals in a catalytically inactive form are good protectants against H_2_O_2_-induced DNA damage (494–509). There are observations suggesting that redox-active metal ions, especially iron, also are important as catalysts enhancing radiation-induced DNA damage (510, 511).

Organic phosphate compounds can form complexes, called perferryl ion complexes, where a ferrous iron ion is coordinated to the negatively charged oxygen atoms in the phosphate group on one side and molecular oxygen on the other (512–517). This binding of an oxygen molecule to ferrous iron is similar to what is found in oxyhaemoglobin. But the oxygen molecule is far more reactive in small complexes of this type than it is in the oxyhaemoglobin molecule (perhaps partly for steric reasons??), even though there are some few substances that can react rapidly with oxyhaemoglobin in reactions leading to methaemoglobin formation (with the iron atom in the haemoglobin molecule being oxidized to oxidation number +3).

It should be noted that the coordination environment of the ferrous iron atom before it binds molecular oxygen is completely different for haemoglobin iron and iron attached to phosphate groups in molecules such as ATP. In haemoglobin, the iron atom is coordinated to 4 nitrogen atoms in planar configuration in the porphyrin ring as well as to an imidazole group below, while the iron atom is coordinated to three negatively charged oxygen atoms when sitting on a phosphate group forming a monoester with some organic molecule. The coordination of the iron atom to several nitrogen atoms in oxyhaemoglobin would be expected to lead to greater stability of ferrous compared to ferric iron in the haemoglobin molecule (because of good binding of ferrous iron to nitrogen), while ferric iron would be expected to be relatively more stable when iron is bound to a phosphate group (because of strong electrostatic binding of ferric iron to the negatively charged oxygen atoms). It is plausible to assume that this will cause the oxygen molecule to be more reactive when bound to a ferrous iron atom coordinated to phosphate on the opposite side than when the ferrous iron atom is coordinated to several nitrogen atoms in haemoglobin, since there will be more Gibbs’ free energy reduction when ferrous iron attached to phosphate is oxidized than when the same happens with ferrous iron in the haemoglobin molecule – which would be expected to lead to corresponding energetic facilitation of a simultaneous (and mechanistically coupled?) 1-electron oxidation of an organic molecule when it it is an oxygen molecule attached to ferrous iron with a phosphate group on the opposite side that reacts both with the iron atom and the organic molecule, compared to what happens in reactions leading to methaemoglobin formation.

Small-molecular perferryl ion complexes can form either directly by reaction between the corresponding ferric iron/phosphate complex and superoxide anion radical, or in two reaction steps where the ferric iron first is reduced by some organic reductant, *e.g*. ascorbate, followed by reaction between the iron atom now in oxidation number +2 and molecular oxygen.

If a ferric iron atom is coordinated to three negatively charged oxygen atoms in a phosphate group, *e.g*. ATP, and also to three negatively charged oxygen atoms in the sulfonic acid group of taurine, these six oxygen atoms will in combination form an octahedron which will shield the iron atom completely from reaction with any other small molecule, including reactive oxygen species (ROS), organic hydroperoxide groups, organic reductants (such as ascorbate), or molecular oxygen (following reduction of the iron atom to oxidation number +2). In the phosphate group, a highly positively charged phosphorus atom in the centre (oxidation number +5) is surrounded by negatively charged oxygen atoms, with the net charge for the whole phosphate group when it it is coordinated to iron (and not forming a double ester as in DNA and RNA) being –2. In the sulfonic acid group of taurine, a highly positively charged sulphur atom (oxidation number +6) is surrounded by negatively charged oxygen atoms, with the net charge for the whole sulfonic acid group being –1. With two negative charges on the phosphate group, one negative charge on the sulfonic acid group and three positive charges on the iron atom, neutral charge balance is obtained, at the same time as octahedral coordination (6-coordination) is the preferred coordination number both for ferrous and ferric iron when found in silicate and oxide minerals in the Earth's crust (518) – for which reason it might be expected that this type of complex might be fairly stable, although far from attaining the stability of chelate complexes, like the ferric iron-citrate complex.

At the same time, it is also reasonable to expect that this type of complex will be sufficiently kinetically labile that it will not hinder the iron atoms from being available for incorporation into iron-dependent enzymes or oxygen-binding proteins such as haemoglobin and myoglobin. Since many of these phosphate-iron-taurine mixed complexes will have low total molecular weight (for the entire complex), much less than for iron-binding proteins such as ferritin and transferrin, they may also be helpful for facilitating the transport of iron atoms by diffusion from one place to another inside the cell (by the principle of facilitated diffusion) in a form where it is not active as a prooxidant catalyst.

In perferryl ion complexes, it is molecular oxygen formally in the same oxidation number as free molecular oxygen which is the reactive oxidizing species. Iron, however, is also very important as a catalyst of reactions involving the reactive oxygen species superoxide anion radical (O_2_^–^) and hydrogen peroxide (H_2_O_2_). These reactions are often referred to as Fenton-type reactions, because it is assumed that hydroxyl radical (OH^–^) formation by reaction between H_2_O_2_ and Fe^+ +^ is an important part of the reaction mechanism when organic molecules are oxidized. This assumption, although one encounters it almost everywhere in articles where biological effects of H_2_O_2_ are discussed, is nevertheless highly doubtful because of the kinetic sluggishness of the Fenton reaction *sensu strictu*, as far as the rate of hydroxyl group formation (by reaction between H_2_O_2_ and Fe^+ +^) is concerned (Dr. Erik Løvaas, Tromsø University, personal communication).

A possible alternative might be a more direct reaction, where H_2_O_2_ itself reacts both with the organic target molecule and with Fe^+ +^ that is complex-bound to the same organic molecule, so that the organic target molecule can be oxidized in a 1-electron reaction at the same time as the iron atom is oxidized from ferrous to ferric. A mechanism of this type might perhaps better explain the sequence specificity of DNA lesions (495) when DNA reacts in metal-catalyzed reactions with H_2_O_2_, compared with the Fenton reaction *sensu strictu* (*i.e*. reaction with hydroxyl radical), which because of the very high reactivity of hydroxyl radical might be expected to lead to a more random attack on any potentially reactive part of the DNA molecule (and not only the most reactive groups) that the OH^–^ molecule first will hit-which is poorly compatible with the observed sequence specificity for the DNA lesions.

Mixed complexes between taurine, iron and a phosphate group on the opposite side may, however, be expected to protect also against this type of reaction, whenever phosphate-bound iron is involved in Fenton-type reactions *sensu latu* (519) – although taurine may not necessarily be any good protectant when an iron atom is bound to other groups than phosphate in the DNA molecule.

It is not unlikely that living organisms also may contain other small organic molecules having a similar function as has been proposed for taurine here, either by helping to form mixed complexes with iron in a way making it catalytically inactive, also when it is bound to groups other than phosphate, or by changing the standard redox potential for the ferrous/ferric equilibrium so much that the iron atoms loses most of its prooxidant activity and instead begins to function more as a protective antioxidant *e.g*. because of superoxide dismutase-like activity of the complex.

Polyamines and trimethylamine oxide (TMAO) might be possible candidates for having an anti-iron protective function in living cells, with the polyamines (because they are already bound to the DNA molecule) perhaps being especially important for protection of the DNA molecule against iron-catalyzed reactions between DNA and H_2_O_2_, while TMAO because of its three methyl groups (which conceivably might facilitate binding of the TMAO molecule to the surface of lipid-rich biological membranes) might be suspected of being especially helpful for reducing the rate of iron-catalyzed peroxidation of lipids and protein molecules sitting in biological membranes. The TMAO molecule contains a semipolar bond between the nitrogen and oxygen atoms, conferring a high negative charge density to the oxygen atom that would be expected to facilitate its binding to ferric iron – at the same time as the 3 methyl groups might be expected to provide effective shielding of the iron atom against contact with ROS, hydroperoxide groups or organic reductants when the iron atom is coordinated to the oxygen atom in the TMAO molecule on one side and either to a membrane lipid molecule or a membrane protein on the other side. Perhaps this form of antioxidative protective mechanism for the TMAO molecule might help to explain the high age normally being attained for sharks (that use a combination of urea and TMAO as osmolytes for attaining the same osmolality as seawater) before they die as a direct consequence of old age.

The polyamines are polycations that may be expected competitively to exclude iron atoms from binding to the negatively charged phosphate groups in the backbone of the DNA molecule, rather than forming mixed complexes where the iron atom is sitting between a phosphate group and the polyamine molecule. But it is not inconceivable that polyamines might participate in the formation of mixed complexes with ferrous iron that is simultaneously bound to a nucleotide base on the opposite side in such way that the ferrous iron atom is shielded against contact with molecular oxygen and H_2_O_2_.

Without knowing the magnitude of the stability constants of any mixed complex between organic phosphate groups, ferrous or ferric iron and the sulfonic acid group of taurine, it would nevertheless appear plausible to assume that such complexes may be fairly abundant when taurine is found at such high concentrations as are common inside most mammalian cells, with exception of erythrocytes (where the intracellular concentration is about the same as in blood plasma, in great contrast to the situation in the nucleated erythrocytes of fishes that contain much taurine), and that this therefore might be an important mechanism helping to explain why taurine functions as an important intracellular antioxidant (520).

It would obviously be desirable to have good measurements of the values for the stability constants of such mixed taurine/organic phosphate complexes both with ferric and ferrous iron. It would also be highly desirable to have more precise information about the stereochemistry of the most abundant complexes between only DNA molecules (and also RNA molecules) and iron, as well as about the stereochemistry of mixed complexes between DNA, iron and taurine (and about the stereochemistry of mixed complexes between RNA, iron and taurine). While there can be little doubt that mixed complexes of this type must be present at much more than negligible concentrations in nucleated mammalian cells (as well as in platelets, which don't have any nucleus, but nevertheless have mitochondria and therefore contain DNA), it is not possible to give any reliable estimate for their quantitative importance as inhibitors of iron-catalyzed oxidation reactions without knowing the stability constants for this type of mixed complexes, combined with what we know about the taurine concentrations in different cell types and different intracellular compartments.

Comparing different types of skeletal muscle in land-living vertebrates, the concentration of taurine is much higher in myoglobin-rich skeletal muscle cells (that also contain much mitochondria), compared to myoglobin-poor muscle cells that contain much fewer mitochondria, but have higher capacity for glycolysis (521, 522). The myoglobin-poor skeletal muscle cells contain instead much histidine dipeptides (522), in humans nearly only carnosine, while in skeletal muscle from poultry both carnosine and anserine are found (523). Carnosine is a good antioxidant, but functions also as an important pH buffer and an important anticarbonyl protective agent (523). The preferential localization of taurine in such muscle cells where ROS production is higher (*i.e*. in cells with much myoglobin and several mitochondria), while carnosine is found in such muscle cells where ROS production is normally much lower, might be taken as an argument that taurine may be a more important antioxidant *in vivo*, compared to carnosine, while carnosine is preferred in such cell types where pH buffering capacity and anticarbonyl protection (because aldehydes, especially methylglyoxal, are formed as byproducts during glycolysis) are relatively more important, compared with antioxidant protection.

It has been proposed that the higher concentration of taurine in myoglobin-rich than in myoglobin-poor skeletal muscle can be explained as a consequence of the localization of much of the taurine inside the mitochondria, where it might possibly also have other functions than antioxidant protection, *e.g*. pH buffering when pH inside the mitochondrial matrix becomes too high because of rapid extrusion of protons to the intermembrane space by the mitochondrial proton-ATPase (522). The taurine concentration may thus be highest in the same parts of the cell where the local H_2_O_2_ concentration in contact with DNA molecules is highest, and where it therefore may be especially important to have high concentrations of such other molecules or inorganic ions (*e.g*. Zn^+ +^ by competitively excluding iron atoms from binding to especially vulnerable parts of the molecule?) that may help to protect the DNA molecules against damage caused by the iron-catalyzed reaction between DNA and H_2_O_2_.

The iron shielding hypothesis presented here is in good qualitative agreement with experimental observations showing that taurine has an antimutagenic effect by protecting DNA molecules against oxidative damage generated by iron-stimulated catecholamine oxidation in the presence of H_2_O_2_ (524): Calf thymus DNA (100 microg/tube) was exposed to a reaction mixture containing: ferric chloride (60 microM), H_2_O_2_ (2.8 mM) and L-dopa (100 microM) (524). Taurine and taurine analogs were added simultaneously with H_2_O_2_ and L-dopa to determine their efficacies in preventing oxidative damage to DNA. The reaction was carried out for 1 hour at 37 degrees C and terminated by rapid freezing in an ethanol/dry ice bath (524). The DNA was precipitated with ethanol and subsequently hydrolyzed with formic acid under vacuum. The hydroxylated bases were separated by HPLC and detected electrochemically. All experiments were replicated a minimum of 5 times (524). Taurine (at a concentration of 20 mM) was found to reduce (p < 0.05) damage to DNA as indexed by reductions in the formation of 5-OH-uracil (49% decrease), 8-OH adenine (37% decrease), and 8-OH guanine (21% decrease) (524). It had, however, only minimal effects on the formation of 5-OH cytosine (<7% decrease) (524). At the same concentration, taurine was also found to increase total DNA recovery after damage by 36–40% and to increase total undamaged guanine by approximately 32% (524). It was found that 5-OH-uracil formation also could be reduced (p < 0.05) by the lower concentration of 1 mM taurine and that 8-OH-adenine formation was reduced (p < 0.05) by 5 mM taurine (524). The effect of some other substances was also tested, and it was found that total base adduct formation was reduced by 20 mM *beta*-alanine (30% decreese), by lysine (58% decrease) and even more by glutathione (88% decrease) (524). When tested at 20 mM, both hypotaurine and homotaurine provided greater protection against DNA damage than taurine, whereas isethionic acid provided a similar level of protection as taurine (524). It may be noted that homotaurine and isethionic acid both contain a sulfonic acid group, like taurine, but homotaurine has greater chain length with 1 carbon atom more than taurine, while isethionic acid has lost the amino group found in taurine and instead contains a hydroxyl group.

The iron shielding hypothesis is also consistent with experimental observations showing that taurine can protect living cells and organisms against toxic effects of iron (39, 181–183), and that it can inhibit iron catalyzed catecholamine autoxidation *in vitro* (525, 526). However, these observations are not sufficient to be taken as proof that the iron shielding hypothesis is correct, and it is not clear if the experimental conditions in the *in vitro* catecholamine autoxidation experiments were similar enough to conditions in living cells that mixed phosphate-iron-taurine complexes could have been formed as abundantly as normally may happen in various intracellular compartments, when the concentrations of taurine and various organic phosphate compounds simultaneously are high. It is not unreasonable that taurine when the concentration is high enough also can form other iron complexes where the iron is rendered catalytically inactive, *e.g*. a complex between ferric iron and 2 taurine molecules (where ferric iron is coordinated to 2 sulfonic acid groups instead of one sulfonic acid group plus one phosphate group).

If the iron shielding hypothesis for explaining much of the mechanism of the *in vivo* antioxidant effect of taurine is largely correct, it means that ascorbate and taurine have complementary roles when they function as antioxidant molecules, since ascorbate is an excellent free radical scavenger (527), but it can also have important prooxidant effects (528–530), which partly may be explained by its ability to reduce ferric iron to ferrous iron, which is far more reactive than ferric iron both in combination with molecular oxygen (512–517) and H_2_O_2_ (519). However, it has been reported that both ferric and ferrous iron must be present if lipid peroxidation shall proceed at a maximal rate (531). By shielding the iron atoms in a way preventing their prooxidant catalytic effects, taurine will simultaneously inhibit those prooxidant effects of ascorbate that depend on reactions between ascorbate and iron, but without inhibiting the antioxidant effects of ascorbate because of its function as a free radical scavenger.

## Antiinflammatory effects of taurine

Following severe radiation injury, it may be expected that an inflammatory reaction will occur, similarly as happens after other forms of tissue injury (*i.a*. as a consequence of large release of proinflammatory substances when cells die in necrosis because of the trauma rather than in apoptosis), *e.g*. because of severe burns (but also following mechanical trauma, *e.g*. severe head injuries), as well as during infection. While inflammatory reactions take place because they have protective functions normally more important than the harmful ones, too much inflammation can easily be harmful and lead to exacerbation of permanent tissue injury following trauma in one form or another, with a typical example being what normally happens following a brain stroke. It is not at all unreasonable that something similar also can happen in various organs following severe radiation injury (with the post-traumatic inflammation causing enhancement of the risk that the patient shall die *e.g*. from vascular shock or from multiorgan failure, and also leading to exacerbation of permanent tissue damage if he survives), and that similar methods that have been found in animal experiments to help to reduce an inflammatory over-reaction (and the permanent tissue injury that it causes), *e.g*. following a brain stroke, also might be valuable for patients suffering from severe injury caused by ionizing radiation.

### Is it possible to treat severe inflammation without suppression of antibacterial and antiviral immunity as a side effect?

Since it is common that severe radiation injury also will lead to immunodepression, at the same time as mucosal organs where viruses or harmful bacteria can easily enter and infect the organism also can be injured as a consequence of too much irradiation, the challenge for the patient's doctors will be the same as when one shall try to reduce the risk that a patient will die from hypervirulent avian influenza, *viz*. to reduce an inflammatory reaction so strong that it is harmful and very dangerous without simultaneously inhibiting too much the organism's immunological defense against viruses and pathogenic bacteria.

What this means is that one must try to weaken so-called neurogenic inflammation (inflammation evoked or strengthened by peripheral nerve cells) and also weaken the activities of such leukocytes that use weapons of so poor specificity that they are capable of inflicting much damage not only to the pathogenic invader, but also to host cells and organs in a way leading to much of what in military jargon is called collateral damage (but in this case to one's own civilian population and not the civilian population of some enemy nation), at the same time as one should also try to strengthen those parts of the immune system that use more precise weapons for targeting the enemy, so that only a minimum of collateral damage to one's own civilian population will ensue. When the enemy soldiers are hiding not in some remote mountain area in Afghanistan, but in the skyscrapers of Manhattan, an American general should obviously stop the over-ambitious colonel who is using machine guns and carpet bombing with dumb cluster bombs killing far more American civilians than enemy soldiers, at the same time as the general might greatly enhance the number of snipers, specially trained commando soldiers and precisely guided drone attacks, as well as trying to do everything else that might be possible for protecting the civilian population. The same principles are also valid if one shall try to save the life of a patient severely affected by hypervirulent H5N5 avian influenza or SARS.

### Parallels between the therapeutic challenges in radiation sickness and hypervirulent avian influenza?

I have earlier written some survey articles together with a nutrition scientist from the Norwegian University of Life Sciences and a physicist from the Norwegian Defence Research Establishment, where we discuss how this hopefully may be achieved in cases of hypervirulent influenza (such as H5N1 avian influenza and the Spanish Flu), as well as about the reasons (with obviously important implications for therapy) why the lethality (risk of dying when one is infected) among patients suffering from hypervirulent influenza is strongly influenced both by the nutritional status (with the average surplus mortality being about 10 times higher in British India than it was in Norway in 1918, and also much higher in other poor countries than in those countries that in 1918 were more affluent) and the age of the patient (due to changes in the secretion of various immunostimulatory and immunosuppressive hormones as a function of age, as well as mitochondrial DNA aging) (520, 532, 533).

While much of what is written about therapy in these articles has never been tested on human patients in well-conducted clinical trials, at least not in form of such multifactorial therapeutic interventions as are proposed in our articles, it may be theoretically expected that if such multiple therapeutic interventions can work for H5N1 avian influenza, they can probably work also for a number of other serious infections (*e.g*. in the hypothetical case that we should get a pandemic with a SARS virus more infective and more lethal than the last one), as well as for patients who have been exposed to high levels of ionizing radiation, when the double challenge is to reduce the level of unspecific harmful post-injury inflammatory response while also trying to reduce the risk that the patient will die from infection as a consequence of the double problem of immunodepression and damage to important mucosal organs.

Taurine can undoubtedly help to improve protection of the “civilian population” (both in the case of hypervirulent influenza and radiation sickness), *i.e*. reduce the risk that the patient will die from a harmful inflammatory over-reaction, because of its antioxidant effect and capacity for scavenging some of those highly reactive antibacterial and antiviral weapons that have low specificity (are imprecisely targeted) and therefore may be especially dangerous to the “civilians”, such as peroxynitrite and hypohalite ions.

Its effects on immunological functions are, however, ambiguous since taurine on one side has been reported to function as a growth factor for leukocytes and very likely also helps to protect them against some of their own weapons, so that they shall not kill themselves too early instead of killing the enemy – but at the same time, it can also function as a negative regulator of inflammatory and immune reactions by mechanisms that will be explained below, and conceivably might weaken not only harmful inflammatory over-reaction, but also useful antibacterial defense functions (although there are good reasons to believe that it is the former function, *i.e*. protecting host tissues by hindering harmful immunological over-response, which is the most important one). Taurine has a number of different functions that all may be expected to contribute to an overall anti-inflammatory effect, especially if it can be combined with other substances that have similar effects and a mechanism of action helping them to interact synergistically with taurine (as might possibly be the case – for reasons that will be explained below-with bromide for patients suffering from severe allergic inflammation), giving a multiplicative effect when these other substances are combined with taurine.

### Reduction of nociceptive pain and neurogenic inflammation because of reduced prostaglandin production and reduced C-fibre sensitization by oxidatively activated protein kinase C

First of all should be mentioned that the well-documented *in vivo* antioxidant effect of taurine (even though the chemical mechanisms explaining it are not completely understood) must be expected to contribute to reduction of prostaglandin synthesis in tissue areas where much damage has occurred, since prostaglandin synthesis is redox-regulated at multiple levels, as has been explained in a recent survey article (534). When better taurine status leads to improvement of the antioxidant defense system of our cells, it must be expected that this also will lead to reduction of prostaglandin synthesis in areas of inflammation because of the combination of less protein expression of cyclooxygenase-2 (COX-2), less oxidative activation of phospholipases liberating precursor fatty acids for prostaglandin biosynthesis, and less oxidative activation of the cyclooxygenase (534). But reduction of the synthesis of prostaglandins in a tissue affected by inflammation means less prostaglandin sensitization of C-fibres (unmyelinated nerve fibres) in this area (532–534). And less C-fibre sensitization means in turn that the release of proinflammatory neuropeptides such as substance P, neurokinin A and calcitonin gene-related peptide (CGRP) from the C-fibres (532) will be reduced. These peptides, because they cause extravasation of plasma proteins through pores that open in the venule walls, are probably important as contributory causes of oedema in the lungs of patients suffering from hypervirulent influenza (532). But it is not unreasonable to believe that they might play an important role in diarrhoeal diseases as well.

The sensitivity of C-fibres is also regulated by protein kinase C (PKC), with PKC activation leading to enhancement of the sensitivity of the C-fibres (533). They contain several different PKC isozymes that all can be activated by oxidative stress (533). There is strong reason to expect that glutathione depletion, *e.g*. in patients suffering from cancer cachexia, will lead to enhancement of C-fibre sensitivity as a result of enhanced oxidative PKC activation, which might in turn lead to intensification of the patient's pain (534). But it is not unreasonable that the activity of PKC in the C-fibres also might be affected by the taurine status of the patient, given the role of taurine as an important intracellular antioxidant.

Since the C-fibres are unmyelinated and very thin (hence having a large specific surface, consistent with their function as chemical multisensors capable of detecting several different abnormalities or normal physiological changes in their local chemical environment), it must be reasonable to suppose that they may have less homeostatic capacity than the brain (which is shielded by the blood-brain barrier) for regulating the intracellular concentrations of nutrient molecules such as taurine, glutathione and ascorbate when the concentrations of these molecules in blood plasma are changed. It is therefore possible that taurine supplementation might help to dampen neurogenic inflammation not only because of reduced prostaglandin synthesis, but also because it may help to reduce oxidative activation of PKC inside the C-fibres.

Another likely consequence of the large specific surface area and lack of myelination in the C-fibres is that they will be much more poorly shielded than cells in the brain to several mutagenic molecules, especially when the latter can act directly as mutagens without prior metabolic transformation (such as ozone in cities with much photochemical air pollutants, acetaldehyde in alcoholics, 4-hydroxynonenal in people eating too much polyunsaturated fatty acids, and inhaled isocyanates, as can happen because of occupational exposure and also happened to a very large number of victims in Bhopal). This may be expected to lead to mutations in the mitochondrial DNA of the C-fibres, which will lead to enhancement of mitochondrial production of reactive oxygen species (ROS) similarly as normal aging does (533), with the ROS coming from the mitochondria next causing oxidative PKC activation, which will lead to enhancement of the sensitivity of the C-fibres (533) and therefore more pain and more neurogenic inflammation, including enhanced risk of asthma (534–534).

There is a good theoretical rationale (as will become even more apparent from the discussion below about the effects of taurine on inhibitory receptors on the C-fibres) for using taurine as part of a multifactorial therapeutic intervention both for Bhopal victims and other patients suffering from disease caused by too many mutations in C-fibre mitochondrial DNA following exposure to high doses of chemical mutagens from whatever source. Even if many of the Bhopal victims are poor and the number of such patients very large, treatment for reducing the sensitivity of the C-fibres in their lower airways should not need to be overly expensive (too much to make it economically feasible), if one uses a combination of enhanced dietary intakes of selenium and other antioxidant nutrients, enhanced intake of long-chain *omega*-3 polyunsaturated fatty acids combined with reduction of the intake of *omega*-6 polyunsaturated fatty acids (especially arachidonic acid if the patient is not too poor to eat much animal foods, but also linoleic acid, being especially important for the poor ones), and high-dose slow-release taurine preparates. Controlled clinical trials for testing the efficacy should, however, first be carried out before applying this form of therapy on the entire affected population in Bhopal.

It would obviously be very useful if similar forms of therapy might be effective also in cities where there is much asthma because of too much air pollution, which is, of course, a major problem in several countries with Mexico City probably being one of the worst examples.

### Cellular damage sentinel function of extracellular taurine

Taurine has multiple physiological roles (520, 532, 535). It is found at much higher normal concentrations (commonly 2 orders of magnitude more) in the cytosol of nucleated cells and platelets than in blood plasma (535). Taurine is therefore a suitable candidate for use as a sentinel of plasma membrane damage or disturbance of plasma membrane functions arising *e.g*. as a consequence of too much oxidative or nitrosative stress, since only a small proportion of the intracellular taurine content leaking out of the cell will be enough to cause a large relative rise in its concentration in the extracellular fluid.

This sentinel function of extracellular taurine can be carried out in two different ways. Extracellular taurine can either bind to receptor sites situated at the outside of the plasma membrane, which may be GABA receptors (which can use either GABA or taurine as ligands) or glycine receptors (which can use either glycine or taurine as ligands), or it can react with reactive halogen species (*e.g*. hypochlorite or hypobromite) that have been formed by microbicidal, viricidal or parasiticidal peroxidases such as myeloperoxidase, eosinophil peroxidase, lactoperoxidase and female genital tract peroxidase that can catalyze the reaction between halide ions (or thiocyanate) and H_2_O_2_ to form anti-inflammatory products such as taurine chloramine and taurine bromamine.

### Roles of taurine and glycine as agonist ligands of inhibitory (hyperpolarizing) glycine receptors on macrophages and neutrophils

Glycine receptors sensitive also to taurine have been found on Kupffer cells, *i.e*. macrophages in the liver (536), where extracellular taurine has an inhibitory action blunting the enhancement of intracellular calcium concentration and TNF-*alpha* secretion following stimulation of the cells with lipopolysaccharide (536). This effect seems to be mediated by a glycine-gated chloride channel (536), so that glycine or taurine binding to the receptor will lead to hyperpolarization of the plasma membrane. It has also been reported that production of TNF-*alpha* and superoxide anion radical (respiratory burst) in alveolar macrophages is blunted by glycine (537). Glycine has been shown to have similar inhibitory effects also in other leukocytes, including neutrophils and lymphocytes (538). Since taurine is an agonist of the glycine receptor in the macrophages (536), it would be expected also to be capable of reducing reactive oxygen species (ROS) production by macrophages both in the lungs, in the liver and in other organs.

Dietary glycine is protective in rat models against tissue damage caused by endotoxemia, liver ischemia-reperfusion, and liver transplantation, and it is believed that this may be mainly explained by glycine inactivating the Kupffer cell via the newly identified glycine-gated chloride channel (538). Similarly, it has been reported that taurine (and also betaine) also protects rats from lipopolysaccharide hepatotoxicity as measured by changes in aspartate aminotransferase and alanine aminotransferase activities and total bilirubin levels in serum, and hepatic glutathione contents (539). Lipopolysaccharide challenge increased serum TNF-*alpha* and nitrate/nitrite in rats, which were reduced by betaine or taurine intake (539). Glycine-gated chloride channels have, moreover, also been found in the plasma membrane of neutrophils, where glycine similarly has been shown to blunt the respiratory burst (540). Taurine should also here be expected to do the same. With neutrophils and monocytes/macrophages both being major players in the pathogenesis of pneumonia induced by hypervirulent influenza, there is good reason to expect that taurine (but also glycine and betaine) might have a similar protective effect in the human lung as has been demonstrated in the liver of experimental animals.

Taurine has also been reported to protect the heart from neutrophil-induced injury during reperfusion following ischemia (213). At a concentration of 15 mM, it was found that taurine markedly reduced luminol-dependent chemiluminescence elicited by activated guinea pig neutrophils as well as by chemically generated hypochlorous acid and hydroxyl radicals, but not by chemically generated superoxide radicals (213). Even though this scavenging effect of taurine for hypochlorous acid and hydroxyl radicals partly may explain why it was protective during reperfusion following ischemia, it must be reasonable to speculate that this was not the whole mechanism, and that glycine receptor-mediated inhibition of the neutrophils also may have played an important role.

Similarly, it has also been found in various studies (244, 246) that an important part of the mechanism for the protective effect of taurine against damage caused by reperfusion following warm ischemia in the liver is a blunting of the activity of Kupffer cells (liver macrophages), leading to reduced phagocytosis (244), reduced leukocyte/endothelium and platelet/endothelium interactions (246) and also being attended by a reduction of the concentration of TNF-*alpha* in blood plasma (244).

### Relevance of inhibitory glycine receptors on macrophages and neutrophils for treatment of rheumatoid arthritis

The glycine receptor-mediated inhibitory effects of taurine and glycine on macrophages and neutrophils might be expected to be therapeutically useful not only for hindering that patients with hypervirulent influenza shall die from asphyxia caused by alveolar oedema (532), but also in such non-infectious inflammatory diseases, *e.g*. rheumatoid arthritis (541–549), where macrophages and/or neutrophils are major players in the disease processes leading to tissue destruction. This might perhaps help to explain why the mother of the present author was permanently cured (for nearly 17 years until she died) for an inflammatory disease in the fingers very similar to rheumatoid arthritis after she had started regularly to eat high doses of fish protein concentrate type B (FPC type B), *i.e*. food-grade fish meal not containing ethoxyquin or other toxic additives, about 50 g per day. FPC type B and fishmeal are good sources both for taurine and glycine, as well as of other substances with anti-inflammatory effects, such as selenium and the long-chain *omega*-3 fatty acids eicosapentaenoic acid (EPA) and docosahexaenoic acid (DHA) (550, 551), and it was because of the selenium content my mother started regularly to take high doses. The diagnosis is not entirely certain since mother was never examined by any rheumatology specialist before she was cured, but she had classical textbook symptoms with the basal finger joints being especially severely affected, which led to considerable deformation of the metacarpal bones that persisted after the active inflammatory process had stopped (and could be seen for all the rest of her life until she died).

### Possible role of necrotic cell death in the pathogenic mechanism of rheumatoid arthritis

It is not well understood why so many macrophages and neutrophils accumulate and become activated in the inflamed joints of rheumatoid arthritis patients. However, mitochondrial DNA and oxidized DNA have been detected extracellularly in the synovial fluid of such patients (552), which can be taken as evidence suggesting that necrotic cell death may be an important part of the disease process (552). It has, however, been reported that mitochondrial DNA can be released from viable neutrophils to form neutrophil extracellular traps, which are extracellular structures able to bind and kill microorganisms (553), so one can not totally exclude the possibility that the mitochondrial DNA that has been found extracellularly in the synovial fluid of rheumatoid arthritis patients might partly or entirely have come from this process, rather than necrotic cell death. It may nevertheless be deemed more plausible, at least as a preferred working hypothesis, that necrotic cell death is the most important mechanism leading to liberation of DNA into the synovial fluid of patients suffering from rheumatoid arthritis.

Mitochondrial DNA is a strong proinflammatory agent (554–556), acting similarly as bacterial DNA via Toll-like receptors (555). Necrotic cell death will occur extensively in affected tissues as a consequence of the disruption of local circulation attending mechanical trauma, and it is also attended by much liberation of other substances with proinflammatory effects (556), such as mitochondrially derived formyl peptides (556–558), cytochrome *c* (559), and high mobility group box protein-1 (HMGB-1) (560–564) (which has a double function both as a nuclear protein and an important cytokine).

Apoptosis is an ATP-dependent process, and necrotic cell death is therefore something that happens when the cell does not contain as much ATP as is needed for apoptosis. An important next question is then what may cause the ATP depletion in cells dying from necrosis in the inflamed joint of a patient suffering from rheumatoid arthritis. It is not implausible that it might be due to a combination of different mechanisms where mitochondrial inhibition by NO (565) and cytokines, such as TNF-*alpha* and IL-1*beta* (566–568), hypoxia, inhibition of mitochondrial enzymes (both in the Krebs’ cycle and the respiratory chain) by too much oxidative stress (*e.g*. happening partly as a consequence of GSH depletion in the mitochondria) and perhaps also depletion of energy nutrients used by the leukocytes (such as glutamine) all may interact synergistically with each other.

### Roles of necrotic cell death and neurogenic inflammation because of excessive C-fibre activation in the pathogenetic mechanisms of lethal systemic responses to extensive tissue injury caused by burns or mechanical trauma: could taurine supplementation be helpful as part of multifactorial treatment strategies for prevention of deaths caused by cardiovascular shock or multiorgan failure following severe trauma?

The extensive liberation of proinflammmatory substances when cells die in necrosis is important for explaining inflammatory responses to injury (556). But is not the only causal factor that is important for explaining the post-injury inflammatory response either locally or at a systemic level, since it is also common that there will be much activation of unmyelinated nerve fibres (C-fibres) transmitting pain signals, and when these C-fibres are activated, they release peptides with vasodilatory and proinflammatory effect, such as tachykinins and calcitonin gene-related peptide (CGRP) (which is a topic that we shall return to below).

If the post-injury inflammatory response becomes too strong, it can locally lead to exacerbation of permanent tissue damage (*e.g*. in form of too much scar tissue development) and at a systemic level to the death of the patient, *e.g*. from cardiovascular shock or multiorgan failure. When an injured patient feels pain, it happens in large measure as a consequence of much C-fibre activation, and it should not be difficult to understand that too much total liberation of vasodilatory and proinflammatory neuropeptides (when the total mass of injured tissue becomes too large, as in patients with extensive burns) easily may have very harmful consequences at a systemic level either as a consequence of too much reduction of total peripheral arteriolar resistance, making it difficult for the heart to maintain the blood pressure (which might be an important part of the pathogenetic mechanism of cardiovascular shock following extensive trauma), or because of too much inflammation leading to too much oxidative stress not only at the injured sites, but also in other parts of the body (which might be important for explaining the pathogenesis of multiorgan failure).

But the post-injury inflammatory response is not only harmful, since some of the substances released because of necrotic cell death and C-fibre activation are very important as positive signals stimulating antibacterial immunity and subsequent tissue repair. The challenge for the doctor is therefore to hinder that the patient shall die from systemic-level consequences of too strong post-injury inflammatory response (because of too large total liberation of proinflammatory and vasodilatory substances from dead cells and C-fibres), while not suppressing too much either the antibacterial immunity or subsequent tissue repair. This is a double challenge very similar to that also encountered in patients suffering from severe radiation injury or severe infections such as hypervirulent avian influenza or SARS. It is therefore likely that similar treatment strategies (where this double objective can be optimally achieved) might be useful for all these groups of patients – which means it probably would also be useful for clinical researchers working with all these apparently very diverse groups of patients to collaborate much more closely with each other than might be common today and exchange experiences, especially about their successes, but also about their failures when trying interventions that did not lead to the expected positive results.

There is also good reason for hope (as hopefully may be apparent not only from what already has been said, but also from the subsequent discussion), that taurine supplementation might be good for all of the patient groups mentioned above (especially when used as part of a more multifactorial therapeutic intervention) as a method of suppressing inflammation that hopefully will not inhibit antibacterial or antiviral immunity too much, will not hinder subsequent tissue repair and also is reported from animal experiments to be antifibrotic (and thus hopefully might help to prevent excessive scar development at injured sites).

### Possible mechanisms of action of fish protein concentrate type B when used as a drug for treatment of rheumatoid arthritis: relevance of glycine receptors on macrophages and neutrophils

When my mother started to take high doses of fish powder (about 50 g per day), it is possible that there may have been an improvement of mitochondrial ATP production leading to reduction of necrotic cell death in the inflamed tissue because of a combination of several different mechanisms:

1. Improved local circulation leading to improved oxygen supply into the inflamed joint tissues (which may have happened partly because of mechanisms explained in references 520 and 534).
2. Reduction of the rate of oxygen consumption by leukocytes for their respiratory burst (because of the combined antiinflammatory effect of various substances found in the fish powder), which must also have helped to reduce the severity of local hypoxia in areas of inflammation.
3. Reduced NO and TNF-*alph*a production (also because of the combined antiinflammatory effect of various substances found in the fish powder).
4. Reduction of the extent of NO mitochondrial inhibition at a given NO synthesis rate because of enhanced glutathione concentration in synoviocytes, leukocytes and chondrocytes leading to improved NO detoxification by formation of *S*-nitrosoglutathione and degradation of this substance (520).
5. Reduction of the extent of H_2_O_2_ inhibition of mitochondrial enzymes in synoviocytes, leukocytes and chondrocytes because of enhanced intramitochondrial GSH concentration and improved selenium status leading to improved scavenging capacity for H_2_O_2_ by the selenium-dependent enzyme glutathione peroxidase, and perhaps also:
6. Improved energy nutrient supply for leukocytes in the inflamed tissue, especially with glutamine which is a very important nutrient for leukocytes, both as a fuel and for other reasons (569, 570).
7. In addition, it is possible that the antiischemic protective effects of taurine (209–265), glutathione and/or glutathione precursor amino acids (312–337), selenium (276–311), all found at high concentrations in the fish powder (550, 551), also may have played a role, helping to reduce the amount of necrotic cell death in the inflamed joints. Since the fish powder my mother took was produced from capelin (*Mallotus villosus*), it must also have contained much anserine, which perhaps may have had an antiischemic protective effect as well, similar to that reported for carnosine (338–346).

My mother was suffering from impaired local circulation in the fingers, causing her fingers to become abnormally cold at low room temperature, but noticed that her fingers became warmer almost immediately after she had taken a large dose of fish powder. The mechanism of this acute effect, which was independently noted by a pharmacologist friend of mine who is now dead (he tried to take a vasodilatory drug for comparison and found that it gave the same effect) and which I can also feel myself, is unknown, but there can be no doubt that there was indeed an improvement of the local circulation in my mother's fingers that preceded recovery from the inflammatory process. It is therefore not at all implausible that improvement of the oxygen supply to her finger joints may have been an important part of the mechanism leading to her recovery.

With less necrotic cell death, there would have been:

1. less liberation of the proinflammatory mediators mitochondrial DNA, formyl peptides, cytochrome *c* and HMGB1, at the same time as the effect of taurine and glycine from the fish powder on glycine receptors in neutrophils and macrophages would have helped to reduce the proinflammatory effect of these substances after they had been released, with these factors in combination leading to
2. further reduction of the local consumption of O_2_ by leukocytes and therefore reduction of the severity of local hypoxia, which would have helped to reduce even more the extent of necrotic cell death leading to liberation of mitochondrial DNA, formyl peptides, cytochrome *c* and HMGB1, as well as to
3. reduction of the production of NO and TNF-*alpha* in the inflamed tissue, which would have led to further reduction of the degree of inhibition of mitochondrial ATP production by NO and TNF-*alpha*, which also would have helped to reduce the extent of necrotic cell death leading to liberation of mitochondrial DNA, formyl peptides, cytochrome *c* and HMGB1.

A positive feedback cycle leading to progressive reduction of the intensity of the disease process might thus have been started, which could explain the process of gradual recovery that happened for my mother over a period of about two or three weeks after the experimental therapy had started, ending with complete cessation of the symptoms of active inflammation and permanent cure.

The reason why this therapy experiment was started was that I had read about antirheumatic effects of selenium in domestic animals in a review article (571) and wanted to try this on my mother, with FPC being the most readily available selenium source for me, since no selenium preparates for use in humans were sold in Norway at that time. But I don't think the highly succesful outcome of the experiment can be explained only as caused by the selenium content of the product (at a dosage level corresponding to about 100 micrograms Se per day). I tried afterwards to persuade a rheumatologist to repeat a somewhat modified version of the experiment (using also some other antioxidants in addition to the fish powder) in a more systematic clinical study, explaining to him what I then thought about pathogenetic mechanisms and possible reasons why the therapy I had tried on my mother did work (which was not the same as I believe today could have been the most important mechanisms explaining the therapeutic effect). He was at first enthusiastic about the idea, and continued to be so also later. But it is possible that he may have got cold feet because some of his colleagues were less enthusiastic, and the clinical study we had been talking about was never started (until finally he died).

Taurine and/or glycine supplementation might presumably be therapeutically useful for patients suffering from rheumatoid arthritis because of their at least partly glycine receptor-mediated inhibitory effect both on macrophages (244, 246, 536) and neutrophils and in the case of taurine possibly also because of its antiischemic protective effect (209–265) helping to reduce the extent of necrotic cell death in the inflamed joints. But because of the pharmacokinetic behaviour of taurine with rapid urinary excretion following intake of high doses, one would presumably need to use a slow-release high-dose taurine preparate for obtaining the desired effect over a sufficiently long period every day.

### Possible relevance of glycine receptors on macrophages and the antiischemic protective effect of taurine for explaining the prophylactic effect of taurine against arterial wall degeneration leading to brain stroke in stroke-prone mice and humans

Another disease where macrophage-induced tissue destruction following severe local hypoxia has been reported to be an important part of the pathogenetic mechanism (572), and where it is possible that the antiischemic protective effect (209–265) of taurine and the glycine receptor-mediated macrophage inhibition by taurine (244, 246, 536) both might be relevant for explaining its protective effect, is cerebral stroke.

It was found in a specially bred strain of spontaneously hypertensive stroke-prone (SHRSP) rats that vascular damage develops due to locally reduced blood supply to the arterial walls in parts of the brain (572). Such vascular damage starts (presumably as a result of severe local hypoxia) at the outer layer of the vascular smooth muscle cells located at the furthest site from the vascular lumen (572). It leads to activation of macrophages in response to the vascular damage, with this macrophage-mediated inflammatory reaction leading to further progression of the vascular lesions, which in advanced stages of the disease also will affect the inner layers of the arterial wall. This may either lead to vascular wall rupture that causes cerebral hemorrhage or to thrombosis inside the damaged blood vessels that causes cerebral infarction (572). The same pathological processes were later confirmed also in human autopsy material (572).

Taurine supplementation was found to have a significant protective effect against this disease process in SHRSP rats (572). It has been proposed that this can be explained by an antiinflammatory effect of taurine mediated by taurine chloramine functioning as an inhibitor of macrophage activation (572). Taurine chloramine is formed by reaction between taurine and hypochlorite, and hypochlorite is formed in a reaction between chloride ions and H_2_O_2_ catalyzed by myeloperoxidase. But since myeloperoxidase is expressed by neutrophils (573) and monocytes (574), while at least in some studies it has been found to be absent from macrophages (573), it is far from obvious that the inhibitory effect of taurine on macrophages in the walls of cerebral arteries can be explained as mediated by taurine chloramine.

It is not unreasonable that the glycine-receptor-mediated inhibitory effect of taurine itself on the macrophages (536) could be more relevant in this particular context, both in SHRSP rats and humans, at the same time as it is also possible that the antiischemic protective effect (209–265) of taurine might be relevant for prevention of the hypoxic damage to parts of the arterial wall that precede macrophage infiltration and activation (and tissue destruction caused by the activated macrophages). If this interpretation is valid, it may be expected that glycine also should have a protective effect (mediated via glycine receptors on the macrophages).

It is, moreover, also possible that other substances than taurine that have been found to have protective effects against tissue damage caused by ischemia and reperfusion in other organs might be protective here as well (because they may help to hinder development of the hypoxic vascular wall lesions that precede macrophage accumulation and activation). It is therefore possible that glutathione and glutathione precursor amino acids (312–337), selenium (276–311), carnosine (338–347) and melatonin (346, 348–474) also might be protective.

### Roles of taurine and GABA as agonist ligands of inhibitory GABA receptors on C-fibres leading to reduction of nociceptive pain and neurogenic inflammation

GABA_B_ receptors have been found on peripheral nerve fibres, both on C-fibres and at the end of cholinergic visceral neurons (575–578). GABA_B_ agonists have inhibitory effects on these nerves, *e.g*. inhibition of release of substance P from capsaicin-sensitive neurones in the rat trachea (578) and of acetylcholine secretion from cholinergic nerves in the lung and in the colon (576–578). GABA itself has been shown to inhibit the anaphylactic response in guinea-pig trachea (579). It has been found that there are different subtypes of GABA_B_ receptors in the central nervous system which differ in their sensitivity to taurine as an agonist (while all of them, of course, are sensitive to GABA). Since the sensitivity of the peripheral GABA_B_ receptors in relation to taurine (whether it functions as a good agonist or not) has apparently not been studied, and it is not known which subtype of GABA_B_ receptors is expressed there, one can not know for certain whether or not taurine may function as a good inhibitor (acting via the GABA_B_ receptors) in relation to these nerves or not. But it is known from animal experiments that taurine and homotaurine have antinociceptive properties in experimental pain models where pain is elicited either by heating (hot plate, tail immersion and tail flick models) or by low pH (injection of acetic acid into the peritoneum) (580–584).

### Anti-asthmatic effects of taurine and taurine-rich fish powder: possible relevance of inhibitory GABA_B_ receptors on C-fibres and parasympathetic cholinergic nerves

Taurine has also been reported to have beneficial effects in an experimental rat model of asthma, where it significantly reduced the number of eosinophils, the lipid hydroperoxide concentration and the Evans blue dye extravasation in bronchoalveolar lavage fluid (585). A therapeutic effect of taurine (when given as an aerosol spray preparation for inhalation) has been reported for human asthma patients as well; it was of comparable magnitude as for commonly used anti-asthmatic drugs (586).

All of this is also highly compatible with my own experience, after I for several years have been taking high doses of FPC type B (about 50 g per day), which as already mentioned is rich in taurine, as a drug for self-medication of acute hayfever and asthma. The effect (*e.g*. reduction of nasal secretion) comes very rapidly, in less than 5–10 minutes, but starts to recede after about 4 hours (which is compatible with what is known about the pharmacokinetics of taurine with rapid urinary excretion when the renal threshold is exceeded).

These observations, when taken in combination, are highly suggestive of an inhibitory effect of taurine on C-fibres, which may in turn lead to reduction of the secretion of proinflammatory peptides such as substance P from unmyelinated peripheral nerves, which means reduction of neurogenic inflammation (even though it could well be possible that there might be more than one pharmacological target explaining the beneficial effect of taurine observed in rats and humans with asthma, so this is not necessarily the only mechanism). It is therefore possible that taurine could be used for reducing C-fibre activity in the lungs and blunting the process of neurogenic inflammation (which possibly might represent an important contributory cause of the alveolar oedema) in patients suffering from pneumonia caused by hypervirulent influenza or SARS. This would be expected not only to lead to reduced extravasation of blood plasma proteins through pores in the venules, as earlier explained, but also less stimulation of leukocytes (*i.a*. macrophages) by peptides secreted from the C-fibres, as well as reduction of the centrally mediated reflex leading to enhanced ACTH secretion (533) and hence enhanced glucocorticoid secretion as a result of enhanced C-fibre activity (533).

However, the effect of fish powder (fish protein concentrate type B, or FPC type B for short) in hayfever and asthma is most likely due not to only to its taurine content, since fish may also be a good source of GABA (that serves as an important osmolyte in fish erythrocytes (587) and probably also in fish skeletal muscle), and one can not exclude the possibility that the product might also contain other substances with antiallergic effect, *e.g*. by functioning as a blocker at histamine receptors. It is also possible that the antiasthmatic and anti-hayfever effects of FPC type B may not only be mediated by an inhibitory action on C-fibres, but also by a direct inhibitory effect on leukocytes, since it can not be excluded that not only neutronphils and macrophages, but also eosinophils and/or mast cells may be equipped with inhibitory glycine receptors, which like the corresponding receptors in macrophages might be sensitive both to glycine and taurine. And the protein found in whole fish must be a good source of glycine, not least from collagen in the fish skeleton. FPC type B is, moreover, also a good source of other nutrients (550, 551) that may be expected to have a more long-term antiinflammatory effect probably relevant for allergic inflammation, such as selenium, glutathione precursor amino acids (plus most likely also glutathione itself, since the product is made from fresh raw material of good quality) and the long-chain *omega*-3 fatty acids eicosapentaenoic acid (EPA) and docosahexaenoic acid (DHA), with most of the EPA and DHA content being in form of membrane lipids.

### Can taurine be used for inhibition of intestinal neurogenic inflammation leading to secretory diarrhoea?

It is not unreasonable that taurine also might have a similar effect in patients suffering from diarrhoea by reducing C-fibre activity in the intestinal mucosa. Substance P is one of the major agents of secretory stimulation in the intestine (588, 589); it will thus stimulate that secretion of chloride ions, accompanied by sodium ions and water across the mucosal epithelial cells into the intestinal lumen, which is an important part of the pathogenesis of diarrhoeal diseases (590). If the secretion of substance P can be reduced by inhibiting the activity of the C-fibres in the intestinal mucosa, this would therefore be expected to help to reduce the chloride secretion and hence the severity of the diarrhoea.

The question might be raised if it is possible for this secretory response to C-fibre activation in the intestine to be anything but harmful to the organism (as is obviously the case when a child dies because of severe dehydration), or stated differently, how it could have helped to enhance the survival probability and thus the Darwinian fitness of our ancestors. One possible answer to this question could be that enhancement of the rate of fluid secretion across epithelial cells lining the surfaces of crypts in the intestinal mucosa may function as a defense strategy for making it more difficult for virus particles to reach the bottom of the crypts and infect the stem cells giving rise to new enterocytes or colonocytes. It is not difficult to understand that it might be useful in a majority of cases if a viral infection can be limited to such cells that even in the absence of infection will only live for a short period before they die and are replaced by new cells, and that it easily might be far worse for the patient if many stem cells also should be infected.

The fluid secretion must then be strong enough to create a current in the central part of the crypt lumen that is faster than the rate of diffusion of virus particles in the upstream direction. This principle can not work just outside the surface of the epithelial cells, where the current velocity in any case must be very low because of fluid adherence to the cell surface (similarly as also happens at the surface of stones lying at the bottom of a swift river). But it might be supplemented by effective killing of viruses in this region, *e.g*. by peroxynitrite produced just outside the surface of the epithelial cells when NO that has been produced inside the epithelial cells by inducible NO synthase reacts with superoxide anion radical that has been produced by an NAD(P)H oxidase located in their plasma membrane. If the C-fibres could not only stimulate secretion, but also might help to enhance peroxynitrite production at the surface of the epithelial cells, *e.g*. by helping to enhance epithelial cell expression of inducible NO synthase, they would by doing so orchestrate a good coordinated defense strategy for minimizing the risk that enterocyte or colonocyte stem cells shall be infected by viruses.

### Relevance of taurine- and/or GABA-mediated inhibition of C-fibres for treatment of rheumatoid arthritis and other chronic inflammatory diseases

It must, moreover, also be expected that C-fibres inhibition by taurine and/or GABA leading to reduced secretion of proinflammatory peptides from the C-fibres may be therapeutically useful for patients suffering from rheumatoid arthritis, psoriatic arthritis or Bekhterev's disease, since these substances will attract, stimulate or activate (at least in non-human species) several types of leukocytes, including macrophages (591–602) and neutrophil granulocytes (603–608). It is therefore reasonable to believe that the combined C-fibre inhibitory effect of taurine and GABA may have been an important part of the mechanisms explaining the therapeutic effect of high doses of fish powder, when my mother was cured from rheumatoid arthritis.

With reduced release of proinflammatory peptides (tachykinins and CGRP) from the C-fibres, it is reasonable to expect that not only the activity of individual leukocytes, but also their total number in the inflamed tissues would have been reduced. The total rate of oxygen consumption by leukocytes in the inflamed tissues must thus have been reduced (by the combined effect of reduced leukocyte numbers and reduced O_2_ consumption per cell), which may have helped to reduce the severity of local hypoxia, at the same time as there would also have been a reduction of the production of proinflammatory cytokines, such as TNF-*alpha*, by leukocytes, again as a consequence both of the reduction of leukocyte numbers and of the activity of individual leukocytes. This would have led to reduction of the inhibitory effect of some of the cytokines themselves on the mitochondrial function of vulnerable target cells (*i.e*. those cell types that express the appropriate cytokine receptors), at the same time as it would also mean less cytokine stimulation of NO production by other cell types, which means that mitochondrial inhibition by NO also would have been reduced.

At the same time, it must also be expected that a reduction of the release of proinflammatory peptides from the C-fibres must have led to reduction of plasma protein extravasation through pores in the venule walls (532), which would mean reduction of oedema and hence reduction of the average diffusion distance for O_2_ molecules between the capillaries and the sites of O_2_ consumption by extravascular cells. The rate of O_2_ diffusion is proportional to the O_2_ concentration gradient, which is decreased (for a given O_2_ partial pressure difference along the gradient) when the distance of diffusion is enhanced. A reduction of the distance of diffusion means therefore enhancement of the rate of O_2_ diffusion from the blood in the capillaries into the surrounding tissue.

This, similarly as for the above-mentioned reduction of the total rate of O_2_ consumption by leukocytes, would also be expected to lead to reduction of the severity of local hypoxia in the inflamed tissue, with the combined effect of improved tissue oxygenation and reduced extracellular concentrations of NO and proinflammatory cytokines (such as TNF-*alpha*) being an improvement of the mitochondrial ATP production capacity of leukocytes and synoviocytes which must in turn be expected to lead to reduction of the rate by which cells in the inflamed tissues die by necrosis rather than by apoptosis. This means in turn reduced liberation of the proinflammatory mediators mitochondrial DNA, formyl peptides, cytochrome *c* and HMGB1 from cells that die by necrosis, and reduced proinflammatory action of these substances – which may presumably interact in synergistic fashion, as far as leukocyte activity is concerned, with the reduced stimulation of their activities by proinflammatory peptides released from the C-fibres because of GABA receptor-mediated C-fibre inhibition.

A reduction of the activity of C-fibres because of reduction of the rate of production of prostaglandins (533–534) or reduced oxidative activation of protein kinase C in the C-fibres because of improved nutritional status with antioxidant nutrients, such as selenium and glutathione (533–534), must be expected to have similar effects as when the activity of the C-fibres is inhibited via GABA receptors, and it is reasonable to expect that all these factors may interact synergistically with each other. The rate of prostaglandin production depends strongly on the fatty acid composition of the diet, being enhanced by a high intake of arachidonic acid and reduced by high intakes of the long-chain *omega*-3 fatty acids EPA and DHA (534). It also depends on antioxidative nutrients such as glutathione and selenium with poor selenium or glutathione status synergizing with high intake of arachidonic acid as causes of enhanced prostaglandin production, which will lead to enhanced neurogenic inflammation (534). At the time when my mother was cured, nothing was done to change her intake of arachidonic acid, but since her meat consumption at that time was fairly modest, it is unlikely that her intake of arachidonic acid could have been very high. The fish powder would, however, have helped to enhance her intake not only of antioxidant nutrients, but also of EPA and DHA.

Trying to explain what happened in patients who have been dead for several years can not be regarded as more than working hypotheses that in principle are not possible either to verify or falsify by such methods that can be used for examining patients still alive. The same will also be the case for patients still alive after the disease has been cured. But if the working hypothesis is a biologically plausible one, it should be possible to test it on other patients still alive. Since rheumatoid arthritis is a severe invalidizing disease associated also with excess death rate, and since it is now often treated with drugs having much adverse side effects, including higly mutagenic cytotoxic drugs (which the present author is convinced should not be used at all except for treating life-threatening conditions when no other efficacious therapy is available), the author would like to express his hope that this might be done the sooner the better, even if it is something going far beyond his own capacity and opportunities as a scientist.

### Possible roles of high blood sugar level leading to enhanced lactic acid production and vanilloid receptor activation as causes of intensification of nociceptive pain and neurogenic inflammation in patients suffering from rheumatoid arthritis and other chronic inflammatory disorders

Another factor very important for the regulation of C-fibre activity is the pH value in the intercellular fluid, acting via pH-sensitive activating receptors such as the vanilloid receptor (also called capsaicin receptor because it is activated by capsaicin, which is the active substance in red pepper – *Capsicum*) (534). The rate of lactic acid production in a porly oxygenated tissue must be limited by the availability of carbohydrate that can be metabolized by the glycolytic pathway to form lactic acid (534). But the quotient between diffusion rates for glucose and oxygen from the blood in the capillaries into the surrounding tissue is linearly proportional to the glucose concentration in arterial blood plasma (534).

It may therefore be expected that a high blood sugar level will lead to enhancement of neurogenic inflammation in patients suffering from rheumatoid arthritis and similar diseases because it leads to enhanced production of lactic acid in the inflamed tissues, which will in turn mean stronger stimulation of the C-fibres via vanilloid receptors. Since I knew nothing about this at the time when my mother was cured (almost 35 years ago), I did not give her any specific advice regarding consumption of carbohydrate-rich foods, which I should certainly have done if I had been aware of it. But she was physically very active in spite of her illness, and she did also eat much vegetables, which are factors that may have helped to reduce her average blood sugar level, in spite of normal consumption of carbohydrate-rich foods such as bread and potatoes.

### Antiinflammatory effects of taurine chloramine and taurine bromamine

Taurine can be halogenated by myeloperoxidase to form taurine chloramine and by myeloperoxidase, eosinophil peroxidase, lactoperoxidase and female genital tract peroxidase to form taurine bromamine, which function as potent anti-inflammatory agents (198–201, 609–616). It is not unreasonable that this also may have been one of the reasons why mother recovered from her rheumatoid arthritis and was completely cured. Taurine chloramine could have been formed because of hypochlorite production by synovial neutrophils and monocytes. I can not reliably estimate the bromide/chloride ratio of her diet, but guess it might have been a little higher than the average for the population in Norway at that time because of abundant intake of vegetable foods, although I don't know if her salt intake was less than average.

Taurine chloramine has been reported to inhibit the production of IL-6 and IL-8 (which is an important chemoattractant for neutrophil granulocytes) by fibroblast-like synoviocytes (609) and also to inhibit the proliferation of synoviocytes (199) in rheumatoid arthritis. It also inhibits inducible nitric oxide synthase and TNF-*alpha* gene expression as well as secretion of the chemokines MCP-1 and MIP-2 in alveolar macrophages (610, 611), which is more directly relevant for patients suffering from hypervirulent avian influenza or SARS, but also may be expected to be highly relevant for patients suffering from rheumatoid arthritis, *i.a*. because reduction of NO and TNF-*alpha* production by macrophages in inflamed joint tissues will help to reduce the risk that cells in these tissues shall die from necrosis rather than from apoptosis. The anti-inflammatory effect of taurine chloramine can in part be explained as a consequence of modification of the inhibitory protein I*kappa*B (202–204), which forms a complex with NF-*kappa*B which must dissociate in order that the transcription factor NF-*kappa*B shall be activated; taurine chloramine will therefore inhibit NF-*kappa*B activation (202–204, 610, 612, 613, 616) with taurine bromamine having the same mechanism of action when this substance also inhibits NF-*kappa*B activation (205). For taurine chloramine, it has been shown that this happens because of oxidation of a methionyl group on the I*kappa*B molecule (202).

Taurine chloramine has, moreover, also been reported to inhibit the activation of Ras following lipopolysaccharide treatment of macrophages and to inhibit ERK1/2 activation in a dose-dependent manner in both RAW 264.7 cells and murine peritoneal macrophages, whereas it did not exert any effect on p38 MAPK activation (616). It has also other protective effects, since HOCl and taurine chloramine may directly neutralize IL-6 and several metalloproteinases in the extracellular environment (615). This may also be expected to be highly relevant in non-infectious inflammatory diseases, such as rheumatoid arthritis, psoriatic arthritis and Bekhterev's disease.

### Possible relevance of low bromide concentration in blood plasma leading to reduced production of taurine bromamine by eosinophils in the pathogenesis of asthma

While myeloperoxidase and possibly female reproductive tract peroxidase are the only mammalian haloperoxidases known to be capable of making hypochlorite from chloride at normal tissue pH values, iodide and bromide have been found to be good reducing substrates (617, 618) for all mammalian haloperoxidases that have been studied. These enzymes can have bactericidal (617), viricidal (619) or parasiticidal (620) effects, and include not only myeloperoxidase, but also lactoperoxidase (617), eosinophil peroxidase (620) and estrogen-inducible (621) female reproductive tract peroxidase. Chloride is also used as substrate for myeloperoxidase, but it is not a good substrate for lactoperoxidase (617) and eosinophil peroxidase (618), except at low pH. Bromide is normally much more abundant than I^–^ in the blood, but the Br^–^ concentration of blood plasma is most likely strongly dependent on the average Br/Cl ratio of the diet. During precipitation of NaCl from evaporating seawater, there is a strong fractionation of Br^–^ relative to Cl^–^ because of the larger ionic radius of Br^–^ (622) compared to Cl^–^. This may explain why the Br/Cl ratio of table salt analysed in Finland (623) was only 1/27 of the Br/Cl ratio found in seawater (622).

In marine aerosols, the Br/Cl ratio is higher than in seawater because of evaporation of biologically formed CH_3_Br from the sea surface (624). The average Br/Cl ratio of rain falling over the continents is therefore higher than in seawater, leading to fairly high Br concentrations in terrestrial plants (623). But there is reason to expect that a large intake of Br-poor table salt may lead to strong reduction of the average Br/Cl ratio of the total diet and most likely also a corresponding reduction of the Br^–^ concentration of blood plasma and other body fluids. It must be expected that depletion of soil Br (and I) as a consequence of deforestation will lead to further exacerbation of this problem.

There are reasons to believe that blood plasma Br^–^ depletion will lead to impaired killing efficiency for all of the microbicidal peroxidases, but more so for lactoperoxidase and eosinophil peroxidase than for myeloperoxidase. Possible consequences might be enhanced risk of mastitis in lactating women (which will enhance the risk of vertical transmission of HIV from mother to child) as well as in dairy cows and goats, impaired killing of tubercle bacilli, and impaired killing of HIV in the vagina before the virus has managed to infect dendritic cells or other leukocytes. However, the kinetic properties of female reproductive tract peroxidase with different halide substrates have apparently never been adequately studied.

In the most common form of allergic asthma (625–628), as well as in other allergic inflammation, eosinophils are major players. In asthma and other allergic diseases, it is therefore the products formed by reaction between taurine and such hypohalite ions that are formed by eosinophil peroxidase that might be most important as anti-inflammatory mediators. Since chloride is of very little importance here, and the production of hypobromite by the eosinophil peroxidase reaction must depend strongly on the bromide concentration in the blood, it may be expected that the reduction of plasma bromide concentrations happening as a consequence of the ingestion of much ordinary table salt may be an important cause of enhanced disease activity (because of reduced production of the inhibitor taurine bromamine) in asthma and other allergic diseases. It may be theoretically expected that a combination of taurine supplementation with normalization of the bromide concentration in blood plasma to the same level as is commonly found in mammals when living in their natural environments might have substantial therapeutic effect by helping to maximize the production of anti-inflammatory taurine bromamine in tissue areas affected by allergic inflammation with eosinophil accumulation and activation.

### Proinflammatory effects of taurine and magnesium depletion and enhanced oxidative stress because of taurine-mediated, magnesium-mediated and oxidative stress-mediated modulation of calcium concentrations in the cytosol of cells participating in inflammatory reactions?

Finally, it is also possible that taurine might exert an anti-inflammatory effect because of the effects of intracellular taurine on calcium transport across various cellular membrane structures (535), most likely because taurine functions as a positive allosteric regulator of membrane electrolyte pumps with only weak binding to the allosteric regulatory binding sites. Since magnesium is needed for the activity of membrane ATPases and intracellular magnesium depletion leads to inhibition of Na^+^/K^+^-ATPase and Ca^+ +^-ATPase activities, taurine depletion will have similar consequences as magnesium depletion for calcium concentrations in the cytosol. The Na^+^/K^+^-ATPase (629–631) and Ca^+ +^-ATPase (632–634) are, moreover, both inhibited by oxidative stress and peroxynitrite, which means impaired transport of calcium away from the cytosol. Cellular depletion of substances important for normal antinitrative or antioxidative defense, such as glutathione or taurine, may therefore be expected to lead to exacerbation of this problem.

But calcium is very important for controlling the discharge of secretory granula into the extracellular fluid. This is also the case when the contents of secretory granula containing substances with proinflammatory effect are released during a disease process attended by inflammation, as exemplified by the release of histamine from mast cells during an allergic reaction. During allergic inflammation, it may, moreover, also be significant that 5-lipoxygenase, which is a rate-controlling enzyme in the leukotriene biosynthesis pathway, is positively regulated by calcium (635). If radiation injury is severe enough to be attended by significant simultaneous losses of intracellular taurine, potassium and magnesium, it may be expected that all these factors may tend to enhance the calcium concentration in the cytosol of leukocytes that can discharge secretory granules containing histamine or other proinflammatory substances at the same time as leukotriene synthesis also might be enhanced.

For correction of these problems, it must obviously be important to try to normalize simultaneously both the intracellular K^+^ and Mg^+ +^ concentrations, intracellular taurine and intracellular concentrations of glutathione and other water-soluble and lipid-soluble antioxidants, such as ascorbate and ubiquinol (coenzyme Q_10_ in its reduced form).

### Roles of ascorbate as reducing cofactor for enzymes scavenging H_2_O_2_ and of coenzyme Q_10_ as an antioxidant

Ascorbate is important for antioxidant defense not only because of its function as a water-soluble free radical scavenger (527), but also because it functions as reducing substrate for the H_2_O_2_- and organic hydroperoxide-scavenging (636, 637) enzyme 1-Cys peroxiredoxin (also called peroxiredoxin-6) (636, 637) and for the H_2_O_2_- scavenging enzyme ascorbate peroxidase. Peroxiredoxin-6 does not only use ascorbate (638), but can also use reduced glutathione (GSH) (636, 637) and dihydrolipoate (639) as reducing cofactors. Peroxiredoxin-6 is expressed (640) very amply (641) in the lungs, and it is also expressed in the mucosa of the mammalian colon (642, 643), in the kidneys (640, 644), in the testicles (645), in oocytes (646) and ovarian cumulus cells (646), and in parts of the brain (647, 648). Many of the reports about this enzyme are, however, from non-human mammalian species, and it is possible that its organ distribution has still not been well enough studied in humans.

Ascorbate peroxidases are heme proteins that have been much better studied in cyanobacteria (649), algae (650) and plants (651) than in animals. However, an ascorbate peroxidase has been found also in the mammalian eye and choroid plexus (652, 653). Its distribution in the rest of the human body, *e.g*. whether it is expressed in other parts of the brain or not, seems to be unknown.

Coenzyme Q_10_, especially in its reduced form ubiquinol, is an important endogenously synthesized lipid-soluble antioxidant (654–658), which because of its location in biological membranes conceivably might be especially important for protection not only of the membrane lipids, but also of membrane proteins against oxidative damage.

### The anti-inflammatory and analgesic effects of taurine could be relevant in a wide range of disease situations ranging from highly lethal pulmonary infections to common pain

The anti-inflammatory and analgesic effects of taurine might presumably be clinically relevant in a very wide range of disease situations (including hypervirulent avian influenza, SARS, diarrhoeal diseases, rheumatoid arthritis, Bekhterev's disease, asthma, painful cancer, common muscle pains such as tension headache, and may be also menstrual pain and pain associated with childbirth), as well as following severe injuries, *e.g*. severe burn injury (in which case there is good reason to suspect that too much neurogenic inflammation with too large total release of tachykinins and CGRP may be a very important part of the pathogenetic mechanism leading to the death of severely injured patients).

Used as an analgesic, taurine has the important advantage that it is almost completely non-toxic and free of potentially dangerous side effects, except for patients with type 1 diabetes (because of a hypoglycaemic effect that possibly might be explained by taurine-induced enhancement of the sensitivity to insulin following its binding to the insulin receptor (659–661), and which might conceivably lead to the development of potentially dangerous hypoglycaemia following insulin injection). This is in great contrast to all currently used NSAIDs, which all can have important side effects (*e.g*. in form of severe gastric hemorrhage induced by aspirin), and also paracetamol, which is mutagenic (534) and greatly enhances the risk of asthma (534), which is a long-term and most likely irreversible side effect that possibly might be related to the mutagenic effect of this drug because of damage to the mitochondrial DNA in C-fibres and other cells in the lower airways, which could lead to enhanced C-fibre sensitivity (because of enhanced oxidative activation of protein kinase C) and in other cell types could lead to enhanced expression of various proinflammatory genes (because of enhanced activity of oxidatively regulated transcription factors, such as NF-*kappa*B) (534).

But if taurine shall be commonly used as an analgesic and as an anti-inflammatory drug, *e.g*. for treatment of asthma (in combination with normalization of the blood plasma concentration of bromide to the same level as is commonly found in wild animals), its rapid renal excretion is a problem, and it would probably be much better if slow-release taurine formulations at a suitable dosage level had been commercially available, rather than using taurine pills or capsules with rapid intestinal absorption.

## Clinical experience using taurine or taurine-rich fish powder for treatment of severe infections (tuberculosis or measles) attended by diarrhoea

There is a very interesting report from the 1930s from a group of Japanese medical scientists (H. Sugihara, S. Nagasawa and H. Okabe) who had studied the effects of taurine both in experimental animals and human patients (662). The report was published in German language in a medical journal in Germany (*Klinische Wochenschrift*), and it is not unreasonable that it may have been overlooked, despite the importance of the reported observations, and later may have become completely forgotten in English-speaking countries, including the United Kingdom and the United States, both as a result of the political situation during the 1930s and World War 2 (which was still remembered in UK and the United States after World War 2 was over, including those serious war crimes that had been committed by medical professionals as part of the genocidal acts of the Hitler regime, especially when carrying out cruel medical experiments on human subjects) and because of the language barrier between German-speaking and English-speaking countries. But this article has nothing to do with those war crimes for which medical professionals both from Germany and Japan were responsible during the 1930s and 1940s; there is absolutely nothing ethically objectionable in it, and it is still well worth reading.

The observations, especially regarding taurine effects on the heart, that Sugihara, Nagasawa and Okabe report from their studies on experimental animals are in good agreement with what other scientists have found in studies on other animal species that have been carried out more than 30 years later. For this reason, it is reasonable to believe that their reports about clinical observations on human patients also may be similarly accurate and trustworthy, even in such cases where the observations have not been independently checked for verification or falsification by other groups of scientists.

After Sugihara et al. had first observed taurine effects on nerve reflexes in animal experiments, suggesting it might have an analgesic effect, they tried to find out if it might also have a similar effect on human patients. They gave taurine in form of intravenous injections at a very high daily dosage level (from 500 mg to several grams per day) to adult human patients suffering from pulmonary tuberculosis and also chronic diarrhoea with severe abdominal pain, most likely as a consequence of intestinal tuberculosis. Following the taurine injection, several of the patients responded by very rapid pain relief, as illustrated by excerpts from the patient journals quoted in the article (662). This also happened in patients who were resistant to the analgesic effect of opiates, but obtained very rapid pain relief following the parenteral administration of taurine. When taurine was given every day, it also had an antidiarrhoeal effect in several patients. But this effect did not come immediately, as with the pain relief, but developed more gradually over some days, perhaps up to about one week after the treatment with taurine had started.

Sugihara et al. report, moreover, also about peripheral administration of taurine, especially for treatment of painful ulcerations (decubitus), where a 0.5% aqueous taurine solution was used, which was applied locally over the surface of the painful ulceration. This did not lead to immediate pain relief as when taurine was given parenterally, but over may be some few hours, excellent pain relief was observed also here. The rapid effect of taurine following intravenous administration and its effect also when applied peripherally must both be regarded as strong evidence suggesting a peripheral rather than a central nervous mechanism for the analgesic effect. These observations by Sugihara et al. are also in good agreement with those animal experiments performed several years later that have demonstrated analgesic effects of taurine and/or homotaurine against pain induced either by hyperthermia (tail flick and tail immersion experimental models) or injection of acetic acid in the peritoneum (580–584).

It is not unreasonable that the antidiarrhoeal effect of high doses of taurine following intravenous injection that was also reported by Sugihara et al. partly might be explained by an anti-inflammatory effect of taurine in the intestine, which might be mediated by a combination of various mechanisms discussed above. However, the time curve for the antidiarrhoeal effect is not easily explained by an anti-inflammatory effect alone. It would be easier to explain the delayed therapeutic response if it might be postulated that it also may have depended on repair of a damaged intestinal mucosa, where reduction of severe inflammation (which might have happened almost immediately after taurine therapy was started) possibly may have been required before the repair process could start, but where it is also possible that taurine may have functioned as a growth factor facilitating rapid growth of enterocyte precursor cells (for reasons that will be explained below) and hence the repair process.

The observations of Sugihara et al. are in good agreement with my own observations from treatment of a large number of patients suffering from the combination of malnutrition, measles and diarrhoea when I was working for the Red Cross in Niger in 1974 (663). About 150 patients were suffering from the combination of malnutrition and measles, and roughly 1/3 of them were suffering from diarrhoea as well. Patients without diarrhoea were given porridge made from a combination of African millet (*Pennisetum* sp.) and fish protein concentrate, type B (FPC type B), *i.e*. food-grade fish meal, that had been produced from capelin (*Mallotus villosus*) that had been caught during the winter season before spawning outside the coast of Finnmark, North Norway. Like most other products made from whole marine fish, not to speak of marine invertebrates such as mussels or squid, FPC made from capelin is an excellent source of taurine.

The porridge was made from 1 part (by weight) FPC and 10 parts unrefined millet flour. The patients were often given extra FPC in pure form, and received also reconstituted milk from skimmed milk powder that had been fermented because of the problem of lactose intolerance, as well as vitamin A in form of red unrefined palm oil (which is an excellent source of *beta*-carotene, *i.e*. provitamin A), and vitamin A tablets for those who had symptoms of vitamin A deficiency. The diarrhoea patients received less porridge than those without diarrhoea, but more fishpowder so that the protein intake should be similar in both groups, and also oral rehydration solution (which was just homemade half-isotonic NaCl solution) and ample well-fermented milk (so as to get rid of as much of the lactose content of the milk powder as possible).

It should be noted that fish protein concentrate apart from its protein (which is of excellent quality in all fish products of good quality) at a concentration of about 70% (of total weight) has a much better composition than myoglobin-poor fish fillet products, such as salmon fillet and cod fillet (550, 551, 623), because of the much higher concentrations of several important nutrients in the fish viscera and skeleton compared to the fish muscle (551), and its mineral nutrient composition is also better than for dairy products and light-coloured meats, such as poultry meat and pork meat (550, 551, 623). It also complements a cereal-based diet as a source of several nutrients that are found at much lower concentrations, are moore poorly available for intestinal absorption or absent in cereals, but which are more typically associated with or better available from animal foods (such as vitamin B_12_, taurine, iron, zinc, calcium, some of the essential amino acids and probably carnitine) or more typically associated with seafoods (such as iodine, selenium, vanadium, long-chain *omega*-3 fatty acids and again taurine).

It should also be noted that cow's milk is a very good source of the intracellular electrolytes potassium, magnesium and phosphorus (623), calculated per g protein in the milk (with the K/protein and Mg/protein ratios substantially higher than in FPC (550, 551, 623) and most other animal foods), which might be important for many patients suffering from the combination of protein-energy malnutrition and diarrhoea, but cow's milk contains very little taurine. Milk is also a better source of riboflavine than fish powder. The concentrations of nucleotides/nucleosides, calculated relative to protein concentrations, were most likely much higher in the fish powder than in unfermented cow's milk, which is not a good source of nucleotides/nucleosides, compared to human milk (664, 665). The fish powder had been produced from capelin (*Mallotus villosus*) that had been caught in the winter during the spawning season. It must therefore have contained much DNA, especially from the gonads of the male fish. It is likely, however, that the nucleotide/protein ratio of cow's milk may be much enhanced during fermentation because of bacterial synthesis of RNA and DNA. The total nucleotide intake (both from the fish powder and the fermented milk) must therefore have been high for my African patients, which is likely to have been helpful both for the recovery of their immune system (664) and for rapid recovery of their intestinal epithelium (664).

Before treatment was started, the death rate had been high; we were told that at the average 2 to 3 patients had been dying every day, and some days up to 5 or 6 patients. But after the combined therapy with oral rehydration and refeeding had started (plus antiinfectious drugs for those who had symptoms of other infectious diseases needing drug therapy), the deaths stopped immediately, and most of the diarrhoeas stopped in only some few days. It is difficult for me to explain the dramatically rapid response to therapy in these patients, unless there has been a very rapid repair of the intestinal mucosa of the diarrhoea patients at the same time as there must have been a very rapid recovery of immunological functions. This should not be impossible, when taking into consideration the very high rate of cellular growth both in the precursor cells giving rise to mature enterocytes and among various classes of leukocytes and leukocyte progenitors, when malnutrition is corrected so that these cells can be supplied with an abundant supply of all nutrients needed for their growth, both essential and conditionally essential ones (such as nucleotides and taurine).

I suspect that the clinical observations of Sugihara et al. from treatment of tuberculosis patients with chronic diarrhoea and my own observations from treatment of patients suffering from the combination of malnutrition, measles and diarrhoea in Africa both might be relevant for treatment of patients suffering from substantial gastrointestinal symptoms because of radiation injury. The doctor should not be tempted to resignate too early when faced with an apparently hopeless situation, following severe radiation injury. And it might presumably be easier both for the doctor and the patient to have some hope, when focussing more on what radiation sickness may have in common with several other forms of disease and injury, as far as the pathogenetic mechanisms as well as repair mechanisms are concerned (and where it is possible, at least in animal experiments and sometimes also in human patients, to achieve very much either for reducing the lethality, as I could observe myself when I was in Africa, or for reducing the severity of permanent organ damage), rather than on the assumed uniqueness of radiation sickness as a foe believed to be so strong that it can not be defeated.

It must be expected, however, that when a patient (or an experimental animal) is taurine-depleted as a direct consequence of exposure to high doses of ionizing radiation, all the anti-inflammatory effects of taurine that have been discussed will be correspondingly weakened, with the taurine depletion itself leading to an enhancement of the strength of the post-injury inflammatory response, leading also to enhancement of the risk that the patient may die not from the direct effects of ionizing radiation *per se*, but because of severe inflammation leading in turn to severe organ dysfunction, *e.g*. in form of diarrhoea.

## Functions of taurine as an osmolyte and as a growth factor that may limit leukocyte growth when the supply is inadequate

One of the earliest studied functions of taurine (587, 666) was its role as an osmolyte important not only for its contribution to the total osmolality of animal cells, but also for cellular volume regulation (587, 666–668). Taurine has later been reported to function as a growth factor for leukocytes or leukocyte progenitor cells (669–673) and apparently also for other cell types including nerve and nerve progenitor cells (674–676), with this growth stimulating effect having been observed even under adverse conditions with hypoxia and reoxygenation (677).

It is not unreasonable that these two apparently completely different functions of taurine might be mechanistically related because of the way by which cellular osmolyte content and volume now has been reported to regulate processes of cellular protein synthesis and apoptosis, at least in liver cells (678–681). The nature of these phenomena, even though it may be possible that they have been well studied only in hepatocytes, suggest that one might be dealing with very fundamental regulatory mechanisms, which presumably also could be very phylogenetically ancient. It is therefore reasonable to believe that they might be found also in all other mammalian nucleated cell types.

It has been found that when alterations of hepatocyte volume are induced either by anisoosmotic environments or under the influence of hormones, concentrative amino acid uptake and oxidative stress function as independent signals which contribute to the regulation of liver cell function and gene expression (681). Several long-known but mechanistically poorly understood effects of amino acids, which could not be related to their metabolism, such as the stimulation of glycogen synthesis or the inhibition of proteolysis, can now be explained as due to their effects on hepatocyte hydration, because they are quantitatively mimicked by swelling the cells in hypoosmotic media to the extent as the amino acids do (681). Likewise, transmembrane ion movements under the influence of hormones have been found to be an integral part of hormonal signal transduction mechanisms with alterations of cellular hydration acting as some kind of "second messenger" of hormone action (681). Integrins act as osmosensors for hepatocyte swelling and trigger activation of mitogen-activated protein kinase systems that function as osmosignaling cascades towards choleresis and autophagy inhibition (681). The opposite process, *viz*. hepatocyte shrinkage, triggers endosomal acidification as a signal for a ceramide-dependent activation of NADPH oxidase isoenzymes, which results in an oxidative stress signal with proapoptotic effects (681). Disturbances of osmosignaling and osmosensing are involved in a variety of pathophysiological conditions such as insulin resistance, protein catabolic states and cholestatic liver injury (681).

It should be added that protein degradation has been found to be heavily redox-regulated in other cell types, such as skeletal muscle cells, with the rate of intracellular protein degradation being enhanced by oxidative stress. When ROS production is enhanced because of NADPH oxidase activation following hepatocyte shrinkage, it is therefore reasonable to expect that this will not only enhance the likelihood that the cell will undergo apoptosis, but also enhance the rate of cellular protein degradation, even in the absence of apoptosis.

There are two different types of protein-degrading multiprotein complexes called proteasomes, *viz*. the 26S and 20S proteasomes. They depend on completely different forms of labelling for finding their target molecules. Protein molecules are targeted for degradation by 26S proteasomes by attachment of a protein called ubiquitin. The synthesis of some of the proteins needed for protein degradation by 26S proteasomes or otherwise is enhanced by the transcription factor NF-*kappa*B (682–684), which can itself be activated by oxidative stress (685–687). Enhanced oxidative stress wil therefore lead to enhanced degradation of muscle protein. NF-*kappa*B activation is involved also in the mechanism of disuse atrophy in the muscle (684, 688). Protein molecules are apparently targeted by oxidative damage for degradation by 20S proteasomes in different types of eukaryote organisms, including plants as well as animals (689–692), while the expression of other autophagy-related genes is enhanced by the oxidatively regulated protein kinase p38*alpha*/*beta* MAPK (693).

The combination of these mechanisms can explain why oxidative stress is a primary trigger (693) of cachectic muscle wasting, but the same signal pathways would be expected to control the rate of protein degradation in other cell types as well. Since it is common that disease situations leading to cachexia not only will be associated with enhanced degradation of protein as such, but also more specifically with enhanced catabolism of sulphur amino acid to form sulphuric acid, there are good reasons to expect that cachexia development easily will be a self-accelerating process once it has started, unless the diet is good enough (with enough sulphur amino acids) to compensate for the enhanced oxidative degradation of sulphur amino acids. One possible reason for this could also be impaired synthesis of proteins that are part of the mitochondrial respiratory chain as a direct consequence of protein malnutrition (533), which will in turn lead to enhanced mitochondrial ROS production because of enhanced Ohmian resistance to the passage of electrons through the respiratory chain (533).

Since taurine is one of the most important organic osmolytes in many cell types (but not universally so), it may be expected that an elevation of the total taurine content of the cell because it helps the cell to expand also may help to stimulate the synthesis of proteins in this cell at the same time as protein degradation and apoptosis will be hindered. At the same time, it may be expected that taurine because of its antioxidant effect (as demonstrated by its antidote effect against several different toxic substances functioning as prooxidants, and also by its protective effect against tissue damage caused by ischemia and reperfusion) will help to counteract cell protein degradation both by 26S and 20S proteasomes, as well as by other autophagy mechanisms.

Again it must be expected that radiation-induced taurine depletion will be harmful because it may inhibit the recovery of organ systems and physiological functions depending on very fast cellular growth, especially in the the immune system and perhaps also in the intestinal mucosa.

### Can the antifibrogenic effects of taurine be useful during treatment of cancer and COPD?

However, opposite effects of taurine on cell growth have also been reported; taurine can sometimes function as an inhibitor of cell growth (694–697), which may probably be explained by its role also as some kind of signal substance inhibiting the growth of certain cell types via receptors located either at the cell surface or inside, perhaps even including nuclear receptors. A more unspecific mechanism caused by its antioxidant effect (and leading to reduced activation of oxidatively regulated signal pathways involving proteins that function as positive regulators of cell growth) can, however, not be excluded, since several different cell surface receptor-associated proteins, protein kinases, protein phosphatases and transcription factors are known which are either directly or indirectly (via proteins that are upstream in the signal pathway) redox-regulated, and which are known to be of crucial importance either for cellular growth regulation or the regulation of apoptosis (so that stimulation of the signal pathways concerned will lead to enhancement of the rate of cell growth and/or suppression of cell death by apoptosis).

Many of these redox-regulated signal proteins (surface receptor-associated proteins, enzymes or transcription factors) are very important in tumour cells, which often seem to exploit them to optimize their own growth and survival by providing an intracellular oxidative or nitrative stress high enough to give good redox-regulated activation of growth-stimulating and antiapoptotic signal pathways, but not so much oxidative or nitrative stress that it directly will cause the death of the cell either through apoptosis or necrosis. Improving the antioxidant or antinitrative defense capacity of the tumour cells compared with this self-sought evolutionary optimum may then paradoxically lead to down-regulation of their growth rate and enhancement of their death rate because of apoptosis, especially when more than one parallel signal pathways can be simultaneously downregulated to obtain a synergistic (multiplicative) interaction, *e.g*. by simultaneous inhibition of several tyrosine kinases (which the present author has tried to exploit for experimental self-therapy, with very satisfactory results, for a disease with marked symptoms suggestive of cancer, but uncertain diagnosis since biopsies have been negative, using very high daily doses during treatment intervals of two days duration of an otherwise very non-toxic substance functioning as a non-specific tyrosine kinase inhibitor, *viz*. genistein). As examples of such directly or indirectly redox-regulated proteins may be mentioned *ras* proteins, several tyrosine kinases, tyrosine protein phosphatases (which in contrast to the corresponding tyrosine kinases are inhibited by oxidative stress), several isozymes of protein kinase C and the transcription factors NF-*kappa*B, AP-1 and Sp1. This topic is much too large, however, that a detailed discussion with literature references is possible here.

Since taurine has been reported to inhibit the growth of cell types such as fibroblasts (695) and vascular smooth muscle cells (694, 697), it might be speculated that taurine supplementation could be helpful in some of those diseases where fibrosis and/or smooth muscle hypertrophy are important parts of the disease process, as in chronic obstructive pulmonary disease (COPD), alcoholic liver disease and other poison-induced fibrosis (in the liver or elsewhere), arteriosclerosis (in which patient group other cardiovascular protective effects of taurine also might be highly relevant), fibrosis in parts of the skeletomuscular apparatus leading to impaired mobility (with fibrosis prevention potentially being a positive side effect if taurine is used regularly for treatment of muscular or joint pains), and fibrosis as a side effect of radiation therapy.

There are already several animal experimental studies, especially with bleomycin-induced (63, 64, 66, 68–72, 75–78) and amiodarone-induced (88) lower airway fibrosis, but also with CCl_4_-induced (30, 141, 144, 147, 148) and alcohol-induced (30, 39) fibrosis in the liver, as well as with cisplatin-induced fibrosis in the kidneys (51), suggesting that taurine may be protective not only in those animal experiments where such an effect has been found, but most likely also in several human diseases attended by fibrosis. These studies suggest that taurine supplementation also might help to protect against radiation-induced fibrosis-which has indeed been demonstrated in animal experiments (9), but might be expected to happen in human patients as well, *e.g*. in cancer patients following radiation therapy.

An obviously relevant question here is how better taurine status might influence the radiation sensitivity of the tumour cells. It is not possible to find out without carrying out the appropriate experiments whether taurine supplementation in cancer patients undergoing radiation therapy really may help to improve the therapeutic ratio (between desired therapeutic effect and unwanted side effects) or not. But it is not a priori implausible that taurine supplementation might be found to be more protective for normal tissues than for the tumour cells. One reason for this is that even if the tumour cells should have good enough uptake capacity for taurine that dietary supplementation will lead to enhanced intracellular taurine concentration and improved antioxidant protection of the tumour cells during irradiation, it must also be expected that enhancement of the taurine concentration inside the tumour cells may lead to reduced activation (as a direct consequence of the irradiation) of oxidatively activated regulatory proteins with anti-apoptotic effect, such as NF-*kappa*B, which will enhance the chance for initiation of the apoptosis programme as a consequence of radiation-inflicted injury to the DNA molecules of the tumour cells (genistein supplementation can probably be used to obtain even more of this effect, similarly as experiments with tumour cells *in vitro* have shown is possible for enhancing tumour cell killing by cytotoxic drugs). And it is not impossible that the last effect paradoxically might be more important than the first one for the overall death rate among the tumour cells following radiation therapy.

A cause-and-effect relationship has been proposed between cellular oxidative damage and increased fibrogenesis based on the fact that experimental treatment with antioxidants either prevents or quenches the fibrotic process (698). With some peculiarities in the different organs, fibrosclerosis is essentially the result of the interaction of macrophages and extracellular matrix-producing cells (698). The cross-talk is mediated by fibrogenic cytokines, among which the most important appears to be transforming growth factor *beta*_1_ (TGF-*beta*_1_) (698). When different types of macrophages are treated with 4-hydroxynonenal, which is a major aldehyde end product of membrane lipid oxidation, this treatment has been found consistently to induce both mRNA expression and synthesis of TGF-*beta*_1_ (698). It is therefore reasonable to expect that taurine also may have an antifibrogenic effect because it functions as an important intracellular antioxidant that helps to reduce the rate of lipid peroxidation and hence the rate of 4-hydroxynonenal production.

Selenium and other antioxidant nutrients would be expected to have similar antifibrogenic effects as taurine and interact synergistically with it because they also will help to reduce the rate of lipid peroxidation and 4-hydroxynonenal production, which will help to limit the expression of the fibrogenic cytokine TGF-*beta*, while a high total intake of polyunsaturated fatty acids (PUFAs) or a high dietary PUFA/oleic acid ratio, other factors being equal, can be expected to enhance the rate of 4-hydroxynonenal production and hence probably will be profibrogenic. It might be speculated that it is not only because of much smoking that there are many COPD patients in Norway, but also because of the average composition of the Norwegian diet with high total PUFA intake, high average PUFA/oleic acid dietary ratio and not especially high average selenium and taurine intakes. It would be interesting to test this hypothesis epidemiologically by comparing the incidence of COPD among smokers (as a function of the total cumulate number of cigarettes) in Norway and Japan.

It is possible that all this may be very relevant for treatment of chronic obstructive pulmonary disease (COPD), if one wants to limit the rate of progression of this disease following early diagnosis. An ideal diet for COPD patients should presumably contain much taurine and much selenium at the same time as the dietary PUFA/oleic acid ratio should be much lower than is common in Norway and several other industrial countries today in order to minimize rates of 4-hydroxynonenal and TGF-*beta*_1_ production. For COPD patients, it is not unreasonable that the antiischemic protective effect of taurine (209–265) also might be highly relevant, if taurine supplementation could help different organs to function better under conditions of tissue hypoxia developing as a consequence of impaired lung function. A cocktail that in addition to taurine also includes other antiischemic protectants such as selenium (272, 276–311) and melatonin (346, 348–474) might possibly be even better for the COPD patient.

At the same time, COPD patients should probably also have a diet rich in alkaline-ash foods in order to compensate both for respiratory acidosis and metabolic acidosis caused by enhanced lactic acid production. This might presumably help to protect them against osteoporosis and reduce the overall level of vanilloid receptor-mediated C-fibre activation, leading in turn to reduction of the reflex-mediated secretion of glucocorticoids that is initiated by C-fibre stimulation (533). With reduction of the total level of C-fibre activation because of higher average extracellular pH, it must, moreover, be expected that there will be less ergoreflex-mediated adrenergic stimulation of the heart leading to reduction of tachycardia, and also less subjective experience of respiratory distress.

Since the sensitivity of C-fibres to stimulation via the vanilloid receptor is enhanced by prostaglandins and by activation of protein kinase C (PKC) inside the C-fibres (533, 534), it will probably also be useful for COPD patients if the diet can be modified so that the rate of prostaglandin production and the degree of oxidative stress-mediated or diacylglycerol-induced activation of PKC inside the C-fibres also can be minimized – for reducing glucocorticoid production, tachycardia and sensation of respiratory distress. This will probably be helpful also for enhancing the subjectively experienced training tolerance of the patient, making it easier to use resistance training for prophylaxis and treatment of COPD-associated cachexia.

To obtain this, the dietary ratio arachidonic acid/(long-chain *omega*-3 PUFAs) should be strongly reduced compared to what is common in several Western countries today, not only by enhancing the intake of long-chain *omega*-3 PUFAs, but also reducing the intake of arachidonic acid at the same time as the intake of antioxidant nutrients including selenium should be high (534). The blood sugar level should be kept down at a moderate level both for reducing lactic acid production in poorly oxygenated tissues (which will directly lead to more activation of the vanilloid receptor) (534) and because it is possible that it may contribute to enhanced production of diacylglycerol in the C-fibres and hence enhancement of the sensitivity of the C-fibres to vanilloid receptor-mediated stimulation (534).

## Safety of taurine preparates

Taurine has been used so much in animal experiments and also as part of the culture medium used for studies on cells *in vitro* (*e.g*. cardiomyocytes) that experimental scientists have gained much experience with the toxicity properties of this substance in non-human animal species and cell cultures. The experience from such studies is that the toxicity of taurine must be very low both for non-human animal species and isolated cells grown *in vitro*.

The safety of taurine for humans can be estimated partly on basis of knowledge about the composition of the diets of hunter and gatherers before the agricultural and industrial revolutions and about the health of such populations, partly from epidemiological studies on the statistical association between taurine intake and health in several different countries today, and partly on basis of reports about possible adverse side effects among people who have ingested high doses of taurine (much above normal intakes) from dietary supplement preparates or “energy drinks” rich in taurine.

There can be no doubt that the taurine intake of hunters and gatherers living on so-called Paleolithic diets (699–704) typically must have been very high, especially for people who have been living in areas with Sub-Arctic or Arctic climate where animal foods were more easily available as food energy sources compared to carbohydrate-rich or fat-rich plant foods such as nuts, root tubers, legume seeds and cereals. It has been estimated that about 2/3 of the food energy intake of hunter-gatherers living in the Kalahari desert typically came from plant foods and 1/3 from animal foods (705), but for Inuits both at the coast and in the inland (Inuit reindeer hunters in Alaska), it must probably have been opposite with at least 2/3 of the food energy intake or more coming from animal foods.

However, it has been common that Sub-Arctic and Arctic hunter-gatherers have been eating much of other plant foods, whatever has been available (including the content of grouse intestines among Inuits in Greenland) (706, 707), which even though they were not very important as sources of food energy still may have been important for the dietary intake not only of vitamin C, folate and polyphenolic plant antioxidants, but also of some mineral nutrients, such as manganese, nickel and copper, that are generally more abundant in plant foods than in animal foods (623), with manganese and nickel being the most extreme examples (623) – with more than 90% of the dietary intake of manganese in Finland during the 1970s coming from plant foods, spices and plant-derived beverages such as coffee and tea (623). It is reported that a group of Inuits living under especially harsh climatic conditions far north in Arctic Canada even were eating grouse faeces (707), which conceivably might have been important to prevent manganese deficiency in a place where little plant foods were available because of the exceptionally harsh climate.

For Sub-Arctic and Arctic hunters, it is reasonable to assume that the daily taurine intake commonly might have been around 1 g per day or more. The general health status of Inuits has nevertheless been good, especially when considering the risk of cardiovascular disease (708, 709), and there is nothing to suggest that their high intake of taurine might have been harmful for their health. Nor is there any suggestion that other “Paleolithic diets” rich in meat, offal and/or seafood and hence rich in taurine have been associated with health problems that might be due to adverse consequences of high taurine intake (699). But there is reason to believe that vitamin D deficiency may have been a severe threat to several populations living in the inland in cold parts of Eurasia during the Ice Age.

In the CARDIAC (CVD and Alimentary Comparison) Study, which was a WHO-coordinated multi-center epidemiological survey on diets and cardiovascular risks and mortalities in 61 populations from different parts of the world, it was found that twenty-four-hour urinary taurine excretion (24U-T) was significantly inversely related with coronary heart disease mortality (572). Higher 24U-T excreters had significantly lower body mass index, systolic and diastolic blood pressure, total cholesterol, and atherogenic index (total cholesterol/high density lipoprotein-cholesterol) than lower taurine excreters (572). Protective effects of taurine on cardiovascular disease risks were found to be intensified in individuals whose 24U-T and -magnesium excretions were higher. One of the groups studied (which was a group of Australian aboriginals with high consumption of seafood) was found to have an average taurine intake during a 2 week study period as much as about 3200 mg per day, far higher than the average for CARDIAC study populations in the rest of the world (572).

It can be concluded from this large study that there is no indication of any health hazard associated even with the highest taurine intake encountered in the study populations (up to as much as 3.2 g taurine per day).

There are, however, some reports about adverse events following consumption of large quantities of taurine-rich so-called energy drinks. It is far from certain that this happened because of high taurine ingestion. There is a possibility that sudden death happened for causes unrelated to energy drink consumption, that it might be related to specific situations in which energy drinks are used, such as ingestion of the energy drink in combination with alcohol (710) or perhaps illegal drug abuse, and also that it may have been caused by too high intake of other substances found in substantial amounts in the energy drink, such as caffeine.

In a recent review article about risks associated with consumption of energy drinks in youths, it is concluded that although the issue of taurine-induced toxic encephalopathy has been addressed, it is likely that the risk of taurine toxicity after energy drink consumption remains low (710). However, whether the prolonged use of energy drinks providing more than 3g taurine daily is safe remains to be examined in the future (710). The consumption of energy drinks may increase the risk for caffeine overdose and toxicity in children and teenagers (710). The practice of consuming great amounts of energy drink with alcohol is considered by many teenagers and students a primary locus to socialize and to meet people (710). This pattern of energy drink consumption explains the enhanced risk of both caffeine and alcohol toxicity in youths (710). 25 to 40% of young people report consumption of energy drink together with alcohol while partying (710). Consumption of energy drinks with alcohol during heavy episodic drinking is associated with enhanced risk of serious injury, sexual assault, drunk driving, and death (710). However, even after adjusting for alcohol consumption, students who consume alcohol mixed with energy drinks had dramatically higher rates of serious alcohol-related consequences (710). It has been reported that the subjective perceptions of some symptoms of alcohol intoxication are less intense after the combined ingestion of the alcohol plus energy drink; however, these effects are not detected in objective measures of motor coordination and visual reaction time (710). The possibility should perhaps not be excluded that caffeine and taurine in combination might lead to reduction of subjectively experienced symptoms of alcohol intoxication sufficiently to enhance the risk of serious alcohol overdose, leading in turn to serious acute disease or death.

When taking into consideration all the observations mentioned above both from experimental studies, epidemiological studies of large human populations and reports about possible adverse side effects of energy drink consumption, it may be concluded that the safety of taurine preparates is very good, and better-as regards the risk of serious side effects-than for a vast majority of commonly used non-nutrient drugs. This is especially important when comparing the potential hazards attending the use of high-dose taurine preparates with such ordinary drugs that are known to have mutagenic effects, such as those cytotoxic drugs that are currently used not only for treatment of otherwise lethal cancer, but also for treatment of the non-lethal disease rheumatoid arthritis, and paracetamol (534), which is regularly consumed at high daily doses, often without prescription, by a huge number of patients all over the world – whereas taurine very likely is antimutagenic for reasons that have been explained in detail above. However, very high daily doses of taurine should not be combined with too much alcohol, too much caffeine or illegal recreational drugs.

It is well known that several other nutrients can have toxic effects when ingested at dosage levels much higher than a normal dietary intake, and one can not exclude the possibility that this might also be the case with taurine, or that there might be paradoxical interaction effects leading to enhanced toxicity of other substances ingested in combination with taurine. Such hypothetical and unknown hazards should, however, be weighted against the known risks or certainly known harmful consequences of the disease or injury where high-dose taurine supplementation might be considered to be used as part of the therapy, and against the possible therapeutic benefits obtained by using taurine.

With something as serious as the health damage caused by excessive levels of ionizing radiation, there can be little doubt what the answer to this question should be (and the same would of course also be the case with life-threatening conditions, such as very serious burn injury, life-threatening asthma, malignant avian influenza or SARS).

There is, however, a potential risk that a sudden large enhancement of taurine intake might have adverse effects in patients suffering from insulin-dependent diabetes. This is because taurine has been reported to bind to the insulin receptor (661) and affect blood sugar regulation, at least in experimental animals (659, 660); there is therefore a possibility more than purely theoretical that administration of high doses of taurine to an insulin-dependent diabetic unfamiliar with the substance might provoke unexpected and potentially dangerous hypoglycemia. The best way to avoid this hazard is probably to reduce the insulin dosage temporarily before starting taurine therapy and subsequently readjust it as required, while keeping the patient under good surveillance and informing him about the possible risk, so that he may modify his carbohydrate intake as needed if he notices symptoms of incipient hypoglycaemia.

## Administration of taurine preparates

Administration of taurine to patients suffering from severe H5N1 pneumonia, SARS or asthma might be done either perorally, by inhalation of an aerosol spray preparate or parenterally. Inhalation preparates have been tried in asthma with good results (586) and would be expected to give an even more rapid effect in the bronchi and lungs compared to peroral administration. But considering the pharmacokinetic behaviour of taurine and its low toxicity, there seems to be no other good reason to prefer inhalation taurine preparates (when considering taurine itself, as different from taurine derivatives that might be degraded in the gastrointestinal tract or liver, and that might be given to the patients for other reasons than because of the effect of taurine itself) rather than peroral or parenteral administration.

For patients suffering from diarrhoea, it should be possible to give them taurine either perorally or by parenteral administration. For conscious patients with a normally functioning intestine, it must certainly be most practical to give this substance by the oral route either alone or in combination with other nutrients or drugs. For unconscious or sleeping patients, parenteral administration should probably be preferred when practically feasible, and the same might also be the case when the intestine is severely damaged in a way leading to malabsorption (even though it is possible that the local effect of high taurine concentration in the intestinal lumen might be useful in some of these patients).

Given the low toxicity of taurine, its numerous protective functions and the facility with which taurine deficiency can develop because of enhanced leakage from individual cells and enhanced urinary excretion in various disease situations or because of therapy (especially during treatment of cancer patients with radiation therapy or cytotoxic drugs), it would probably be right to add taurine as a matter of routine to preparates for parenteral nutrition, as well as to food preparates to be given through nasogastric tube. The reported antidiarrhoeal effect of taurine (662), which is consistent with the very rapid recovery of the patients observed by the present author when using a diet rich in taurine in combination with oral rehydration solution for treatment of a large group of children suffering from the combination of malnutrition, measles and diarrhoea in Africa (663), needs further testing in animal models as well as in human patients. But if this effect can be confirmed, it may be possible that it could also be therapeutically useful to add taurine to preparates used for oral rehydration of cholera or other diarrhoea patients.

The pharmacokinetics of this substance with normally good intestinal absorption, but rapid urinary excretion (when it is given at a high dosage level) should be kept in mind; for oral administration of taurine preparates (being perhaps not equally important when abundant taurine is given as part of a food mixture with enough bulk that it will not pass too rapidly through the stomach into the intestine), it might therefore be useful to have a slow-release formulation securing that there will be a high plasma taurine concentration for a more extended period of time. Such formulations are perhaps not commercially available in any country in the world today, but WHO and the health authorities of individual countries should encourage pharmaceutical companies to make them. The dosage level for severely ill adult patients should probably not be less than about 1 g taurine per day. Taurine appears to be exceptionally non-toxic, which means that the risk of harmful side effects may be judged very small, except possibly for insulin-dependent diabetics, for reasons which earlier have been explained.

## Taurine should not be used alone, but as part of a multifactorial strategy for treatment of severe radiation injury

In all of its known physiological functions, taurine collaborates with several other nutrients and/or hormones. Several other dietary deficiencies can therefore mimick taurine deficiency in some of their functional consequences, and the antioxidative protective hormone melatonin can mimick some of the positive therapeutic effects of taurine supplementation, not only for protection against tissue injury caused by ischemia/reperfusion (346, 348–474), but also for protection against ionizing radiation (711–771). For obtaining an optimal therapeutic response following severe radiation injury, it will not be helpful to think of taurine as some kind of “magic bullet” that can be used alone, in spite of numerous different beneficial effects. Rather, one has to combine everything that is needed for optimizing some particular physiological function (or even for allowing it to occur).

Protective effects during or following exposure to ionizing radiation have been reported also for selenium (772–794), glutathione and glutathione precursors (795–806) and carnosine (807, 808), which are substances that earlier have been mentioned as examples of antioxidative nutrients that offer significant protection against tissue damage caused by ischemia and reperfusion, similarly as has been found for taurine in a large number of experimental studies. However, not all reported observations are consistent regarding radioprotective effects of glutathione (which apparently depend on experimental conditions and are not always observed). Carnitine and carnitine derivatives (809–816), and numerous plant antioxidants (literature so abundant that references are omitted here, but it can easily be found on PubMed) have also been reported to function as radioprotectants.

The radioprotective effects of antioxidant nutrients and the antioxidant hormone melatonin can in large measure be explained by their effect on the cellular capacity to scavenge harmful reactive species that are formed because of the passage of ionizing radiation through cells (817), such as hydroxyl radical (OH^–^), peroxyl radical (HO_2_^–^), superoxide anion radical (O_2_^–^). hydrogen peroxide (H_2_O_2_), singlet oxygen and peroxynitrite (475). Some of these radioprotective substances, such as selenium and glutathione, act mainly via antioxidant scavenger enzymes, such as glutathione peroxidases and peroxiredoxins (817). Carnosine (818–825), melatonin (713–715, 719–721, 727) and numerous plant-derived antioxidants (826) function themselves as scavengers of some of those harmful molecules that are produced either more directly by ionizing radiation (such as hydroxyl radical and peroxyl radical) or by radiation-induced lipid peroxidation, including organic free radicals and in the case of carnosine such reactive carbonyl compounds that can be formed as secondary products of lipid peroxidation (523), with carnosine (818–820) and melatonin (713–715, 719–721, 727) both being good scavengers of hydroxyl radical and peroxyl radical.

Melatonin has, moreover, also been reported to scavenge superoxide anion radical, singlet oxygen, and peroxynitrite (720), and carnosine is reported to function as a scavenger of peroxynitrite (827), which is formed when peroxyl radical or superoxide anion radical produced by radiation reacts with NO that has been produced by NO synthases in radiation-exposed cells or some of their neighbours (because NO is very freely diffusible and can diffuse over some distance from one cell to another before it is scavenged). Only poor scavenging of peroxynitrite by carnosine was, however, found in another study, where, nevertheless, carnosine was found to be a good scavenger of hypochlorite (828), which may not be relevant for radiation injury, but could be so for non-infectious inflammatory diseases, such as rheumatoid arthritis and asthma. Carnosine has, moreover, also been reported to function (similarly as glutathione also does) as a scavenger of NO (829, 830), which will help to reduce the rate of peroxynitrite production, while melatonin reduces the rate of NO formation by reducing the activity of neuronal (831–834) and endothelial (835) NO synthase. This happens apparently because melatonin binds to calmodulin (832, 833, 836) and hence either modifies the stability of the activated (NO-producing) complex between a calcium-calmodulin complex (containing 4 Ca^+ +^ ions) and the enzyme itself (which possibly could mean that a higher calcium concentration might be needed for activation of the enzyme) or changes the conformation of this active enzyme complex in a way leading to reduction of the activity of the calcium/calmodulin-activated enzyme (in which case the calcium concentration needed for activation might remain the same, while the effect of calcium influx into the cell on NO production nevertheless will be reduced because the kinetic properties of the active enzyme have been changed). Melatonin exerts a similar regulatory effect also on at least one of those other enzymes that are activated by the calcium-calmodulin complex, *viz*. Ca^2 +^/calmodulin-dependent protein kinase II (837). Peroxynitrite reacts with DNA molecules without any need for assistance from iron or other redox-active transition metals that function as catalysts of DNA oxidation by H_2_O_2_. It is a powerful mutagen which oxidizes DNA bases and can produce both single- and double-stranded breaks in the DNA molecule (838–843).

Yet another class of radioprotectant molecules (probably including taurine and the histidine-containing dipeptides carnosine and anserine) may work by counteracting the prooxidant catalytic effect of iron or other redox-active transition metals. But carnosine and taurine have a double mechanism of action for their radioprotectant effects since they also function as scavengers of some of those reactive species that can react with DNA molecules or enhance the rate of lipid peroxidation.

Melatonin, when acting as a hormone via nuclear receptors, helps, moreover, also to enhance the expression, at least in some cell types and organs (such as the brain), of several enzymes that either function as antioxidant scavengers themselves (such as superoxide dismutase and glutathione peroxidase) or participate in the electron transport chain needed for the function of some of the scavenger enzymes (such as glutathione reductase and glucose-6-phosphate dehydrogenase, which in most human cells are needed for normal regeneration of GSH from GSSG in the cytosol and hence are required for the function of glutathione peroxidase in the cytosol) (715, 719).

It must be expected from this that there will be a number of synergistic (multiplicative) interactions between various antioxidant nutrients and melatonin when functioning as radioprotectants. Selenium and glutathione will interact synergistically because of the tert-uni ping pong kinetics of glutathione peroxidases (534), that scavenge H_2_O_2_, organic hydroperoxides and peroxynitrite (534), and melatonin will interact synergistically with these antioxidant nutrients when it enhances the expression, at least in some organs, of glutathione peroxidase, glutathione reductase and glucose-6-phosphate dehydrogenase.

For peroxynitrite there will also be two other effects of melatonin interacting synergistically with the above-mentioned one: when it reduces NO production by neuronal and endothelial NO synthase and when it functions itself as a scavenger of peroxynitrite. When melatonin has multiple different effects helping to reduce the concentration of peroxynitrite in cells expressing neuronal NO synthase, it means that the dose-response curve for peroxynitrite concentration in brain cells as a function of melatonin concentration in blood plasma will be strongly nonlinear. There is reason to believe that this kind of non-linear dose-response curve (which will help this hormone to function much like an on-off switch) may be very important for understanding the effects of melatonin as a regulator of diurnal biological rhythms.

The non-linear off/on switch-like shape of this curve suggests also that the concentration of peroxynitrite in brain cells may be one of the most important targets of melatonin when acting as a regulator of diurnal rhythms. One possible explanation might be that peroxynitrite (which will be produced abundantly in brain nerve cells during the day following activation of NMDA receptors) could be critically involved in learning processes as one of the most important signal substances that start a chain of processes that will initiate subsequent protein synthesis needed for those microstructural anatomical changes (with the synaptic contacts between some pairs of nerve cells being improved because of enhancement of the total number of synapses between the two nerve cells concerned) that are needed for establishment of long-term memory. But since peroxynitrite has also a number of harmful effects to the brain cells, a prolonged period with little peroxynitrite during the night might be needed for repair of those peroxynitrite-induced lesions that have accumulated in the brain during the preceding period of light, wakeful sensation and learning the day before.

Glutathione will also help to reduce the rate of peroxynitrite production because of its reaction with NO leading to *S*-nitrosoglutathione production, with *S*-nitrosoglutathione not forming peroxynitrite when it subsequently reacts with superoxide anion radical (520), and taurine will probably interact synergistically with H_2_O_2_-scavenging enzymes when protecting against the catalytic effect of iron against reactions between H_2_O_2_ and organic molecules.

The theoretical rationale for using a cocktail of different antioxidant nutrients and melatonin for obtaining optimal radioprotection rather than using high doses of only one of these substances alone would thus appear very strong. It is possible that this could have important practical implications not only for radioprotection following severe nuclear accidents, but also for reducing the side effects from radiotherapy in cancer patients – especially if this principle could be used for protecting normal tissues at the same time as other methods (*e.g*. inhibition of polyamine synthesis in the tumour cells while supplementing the patient with enough polyamines to maintain normal levels in the normal tissues (817)), are used for selectively enhancing the radiosensitivity of the tumour cells.

Another good reason for preferring multifactorial treatment strategies to monofactorial therapeutic intervention (say, with high doses of one drug) is also that a good multifactorial treatment strategy often will be associated with much lower risk of adverse side effects than when high doses of only one pharmacologically active substance are used. This is because the different substances that are used as components for multiple intervention very commonly will have different side effects, even if they collaborate in synergistic fashion to produce the same desired therapeutic effect, and sometimes it is even possible that some of the substances that are used for the multiple intervention can protect against the side effects of other substances that are also used as part of the same multiple treatment strategy (534). When different substances interact synergistically with each other to produce the same therapeutic effect, it is possible to reduce the dosage level for each one of them with corresponding reduction of the risk of adverse side effects, and still obtain the same desired overall therapeutic response (or even a better one). Moreover, it is also common that nutrients, when used as part of multiple treatment strategies, are less toxic and therefore associated with less risk of adverse side effects (even when given at doses higher than normal dietary intakes), compared with most ordinary drugs. The same is also the case with the antioxidant and immunostimulating hormone melatonin and some of those other substances that can be used for immunostimulation.

From a theoretical standpoint there must therefore be very good reason to hope, for several different diseases and severe injury conditions, that well-designed multifactorial treatment strategies incorporating optimal nutrition as an important part of it might be able to improve the therapeutic ratio very substantially (with significant improvement of the therapeutic outcome at the same time as adverse side effects can be reduced) compared with such monofactorial or oligofactorial treatment methods that are used for the same diseases and injuries today (534). And they should in a majority of cases not need to be overly expensive (since some of those substances that often might be used can be produced very cheaply and are not protected by patents), which means that they also as regards their health economic cost-benefit ratio might be very favourable, making it possible for a society with limited economic and skilled manpower resources to take care of a larger number of patients than today at the same total cost, and yet achieve better average therapeutic results than now. While this is obviously even more important in poor countries than in the more affluent ones, the problem of resource constraints compared with the number of patients needing medical treatment and care is in principle the same almost everywhere, even in rich countries such as the United States and Norway, with only some very few possible exceptions.

There are two different (each of them very strong, but even more compelling when seen in combination with each other) reasons for confidence that the results of experiments for studying the radioprotective effect of individual substances mentioned above in other mammalian species can safely be extrapolated to humans. The first is that one is dealing with very fundamental physical, chemical and biochemical mechanisms that must be expected to be the same everywhere in all living cells and organisms that use the same set of antioxidative protective enzymes as we do. The other is the same as has been mentioned when discussing the evidence for protection by antioxidant nutrients and melatonin against permanent tissue damage caused by ischemia followed by reperfusion, *viz*. the combination both of the quality and abundance (*i.e*. number of independent studies) of animal experimental observations showing essentially the same when confirming the radioprotectant effect. The number of independent experimental studies is also in this case (as for antiischemic protection) especially large for melatonin.

It is possible that the inconsistent results of experimental studies of the radioprotective effect of glutathione partly or wholly might be explained by diverse effects of glutathione both as an antioxidant and a prooxidant, with not all effects being protective when a cell is exposed to ionizing radiation. Reduced glutathione (GSH) must be expected to be protective because of its function as reducing cofactor for H_2_O_2_-scavenging, organic hydroperoxide-scavenging and peroxynitrite-scavenging antioxidant enzymes such as glutathione peroxidases and peroxiredoxin-6 (1-Cys peroxiredoxin), and also by functioning as a complexing agent for ferrous iron (with the GSH/ferrous iron complex being, if not necessarily directly protective, at least far less active as a prooxidant compared to several other complexes between ferrous iron and small organic molecules, including the glutathione degradation products cysteinylglycine and cysteine). But GSH may also be expected to have an adverse effect, similarly as vitamin C does, by functioning as a reducing agent for ferric iron that is complex-bound to other organic molecules (although it is possible that the harm resulting from this effect may be far less for GSH than for ascorbate because of higher stability of the complex of ferrous iron with GSH than for the complex between ferrous iron and ascorbate).

However, it should in theory be possible to exploit the synergistic interactions between glutathione and other radioprotective substances to enhance its beneficial effects and reduce the possible harmful ones. If the levels of glutathione peroxidase and associated enzymes (such as glutathione reductase and glucose-6-phosphate dehydrogenase) can be enhanced by supplementation with selenium and/or melatonin, this would be expected to enhance the protective effect of glutathione when it helps to scavenge H_2_O_2_, organic hydroperoxides and peroxynitrite, and it is possible that taurine could be used for reducing its harmful effect when GSH functions as a reductant for ferric iron.

It may be noted that most species of seafish are good sources not only for taurine (550) but also for selenium (550, 551, 623), for glutathione precursor amino acids (550, 551), and when the fish has been freshly caught presumably also for glutathione itself. Histidine dipeptides, especially anserine, which has been very little studied but appears to have similar effects as carnosine as a radioprotectant (807), are found at very different concentration levels in differrent species of fish, but some species, *e.g*. capelin (*Mallotus villosus*), contain much anserine (844–849). Since sea fishes, in great contrast to humans and several other mammalian species living on land, do not normally eat much starch or sugar, it is common that they rely on protein and fat as their most important metabolic fuels. It should therefore be expected that pelagic fast-swimming species with good aerobic capacity must have large capacity for *beta*-oxidation of fatty acids, which means that they must contain much carnitine in order to get the fatty acids transported fast enough into their mitochondria (and probably even more so when the water temperature is low). Although it is difficult to find analytical data for carnitine concentrations in different types of fish and different fish organs, it may be expected that many fish species are excellent dietary sources of carnitine, with the concentration perhaps being especially high in pelagic species living in cold water, such as capelin, as well as in the fastest swimmers, such as tuna.

It may be concluded that the average intake of radioprotective nutrients probably may be higher in Japan than in most other countries in the world because of exceptionally high average seafood consumption, at the same time as the average intake of plant-derived antioxidants also is high in Japan (*i.a*. from soy products and tea). It is probable that this may have helped to reduce the health damage caused by high doses of ionizing radiation among survivors from Hiroshima and Nagasaki, and there is also good reason to expect that the composition of the normal diet in Japan is protective today for those who have been exposed to high levels of ionizing radiation while working with damaged nuclear reactors. But it is possible that this form of protection may be further improved by additional taurine supplementation, and there is in particular reason to expect that it might be improved by melatonin supplementation at a high dosage level (*e.g*. 20 or 40 mg melatonin per day for adult patients).

For people living in countries where seafood consumption is not as high as in Japan, FPC type B combined either with antioxidant-rich plant foods or plant antioxidants given as dietary supplements would probably be excellent for enhancing the total dietary intake of radioprotective antioxidative nutrients. The FPC dosage level for adult patients suffering from radiation injury should probably not be less than 100 g per day.

### Nutrients needed for DNA replication and repair

Normal cell growth cannot happen without an adequate supply of several different molecular building blocks, both macronutrients and micronutrients, and not only essential ones but also conditionally essential nutrients such as taurine and nucleotides. This is the same both when considering the growth of enterocyte progenitors and that of several different classes of leukocytes or their progenitor cells.

DNA repair is also a process dependent on several different nutrients (but apparently not, as far as is known, taurine). They are in large measure (since DNA repair cannot proceed normally without an adequate supply of monodeoxyribonucleotide building blocks) the same ones that are also required for DNA synthesis and therefore are needed for fast cellular growth, which in turn is absolutely required for fast recovery both of the immune system (if it has not been damaged beyond repair) and of the intestinal mucosa (again if it has not been damaged beyond repair). But DNA repair depends also on other enzymes that do not participate in DNA replication, and some of these other enzymes (that are needed for normal DNA repair) depend also on nutrient cofactors making them potentially vulnerable to malnutrition.

For minimizing the harmful biological effects of ionizing radiation by interventions carried out before, during or after exposure (*e.g*. before people shall go into a severely damaged nuclear power plant in an effort to try to hinder further escalation of the nuclear disaster), it will be helpful to try to do everything possible for optimizing antioxidant protection (not only during the exposure to radiation, but also afterwards in order to minimize tissue injury caused by the post-exposure inflammatory response) and also for DNA repair processes (both during and after the radiation exposure). After exposure to ionizing radiation has occurred, it will be important to continue to maintain an optimal supply of all nutrients that are needed for normal DNA repair. When much DNA damage occurs, this would be expected to lead to acutely enhanced demand for all mononucleotides that are needed for the DNA repair process, perhaps even so much as to induce mononucleotide deficiency that might limit the rate of normal DNA synthesis and cellular growth – which could represent a possible explanation both for immunosuppression and gastrointestinal damage following exposure to high doses of ionizing radiation.

The dietary requirement for nucleotides/nucleosides is therefore likely to be higher in patients who have suffered from severe radiation injury than in healthy persons, perhaps by a significant factor, at the same time as intestinal injury conceivably might be attended by malabsorption making it more difficult to absorb as much nucleotides or nucleosides that may be needed to allow both optimal DNA repair and optimal cell growth, *e.g*. in leukocytes and leukocyte progenitor cells. Intestinal malabsorption might, of course, also affect the absorption of nutrients needed for endogenous synthesis of deoxyribonucleotides by *de novo* pathways, such as glutathione precursor amino acids or glutathione itself (which participates in deoxyribonucleotide synthesis because it is needed as part of the electron transport chain from NADPH via glutaredoxin to ribonucleotide reductase), riboflavin (which is needed in both parallel pathways for electron transport from NADPH to ribonucleotide reductase either via glutaredoxin or thioredoxin), thiamine (which is needed for normal regeneration of NADPH from NADP^+^ because of the role of thiamine pyrophosphate as a cofactor in transketolase), folate (which is needed for conversion of uridylate to thymidylate) and vitamin B_12_ (which is also needed for conversion of uridylate to thymidylate).

If the cells contain too little deoxythymidylate when they shall synthesize or repair DNA, deoxyuridylate will be incorporated into the DNA molecule instead of deoxythymidylate, *i.e*. the DNA molecule will now contain uracil in what should normally have been a thymine position (850–853). This is a form of DNA damage that can be repaired, but the repair mechanism does not function perfectly, especially not when thymidylate continues to be deficient, and it may sometimes lead to DNA double-strand break instead of correct repair (850–852). This can happen not only because of folate deficiency (850, 854), but also because of vitamin B_12_ deficiency (854–856). A similar effect can, moreover, also be expected to occur because of zinc deficiency, since thymidine kinase, which is an enzyme needed for reutilization via the salvage pathway of thymidine for making DNA, is a zinc-dependent enzyme vulnerable to zinc deficiency (857).

It must be expected that there will be a synergistic interaction between zinc deficiency and low dietary intake of DNA as causes of reduced thymidylate synthesis by the salvage pathway, with the mutagenic effects of thymidylate depletion being especially severe when thymidylate synthesis both via the salvage and *de novo* pathways are simultaneously inhibited *e.g*. because of a combination both of zinc deficiency and vitamin B_12_ deficiency. This is something that easily can happen when the total dietary intake of animal foods is low (551) at the same time as the local agricultural soils are depleted in zinc because of the combined effects of prolonged chemical weathering under humid tropical conditions and deforestation (551, 858), which is a situation very commonly encountered in much of sub-Saharan Africa.

It can, moreover, be expected that there will be a synergistic interaction between those dietary deficiencies that specifically affect the synthesis of thymidylate and such dietary deficiencies that will cause reduction of the activity of ribonucleotide reductase and hence may lead to reduction of the synthesis of all deoxyribonucleotides needed for DNA synthesis and repair. In addition to those nutrients that are needed for normal electron transport to ribonucleotide reductase via glutaredoxin and already have been mentioned (glutathione and its precursor amino acids, riboflavin, niacin and thiamine), iron is also needed here because ribonucleotide reductase itself is an iron protein (which uses iron as a necessary catalytic cofactor), and selenium is needed because thioredoxin reductase (which is needed for electron transport to ribonucleotide reductase via thioredoxin) is a selenoprotein (859). The combination of dietary deficiency of sulphur amino acids and selenium, which might be geographically widespread in sub-Saharan Africa (551, 858), must therefore be expected to be strongly unfavourable, as far as DNA repair is concerned, and the situation will not be improved by simultaneous deficiency of zinc, iron or B-group vitamins also needed for normal DNA synthesis and repair.

Niacin and tryptophan are important for DNA repair not only because of the role of NADPH in both of the two parallel chains of electron transport leading via glutaredoxin and thioredoxin to ribonucleotide reductase (since thioredoxin reductase, like glutathione reductase, is a flavoprotein using NADPH as reducing cofactor), but also because of the role of NAD^+^ as substrate for poly(ADP-ribose) polymerase, which is an enzyme needed for normal DNA repair *i.a*. because it functions as an intracellular sensor for DNA strand breaks (860–862). The combination of niacin and tryptophan deficiencies deficiencies leading to NAD^+^ depletion (863) is therefore mutagenic (864, 865), which should give strong reason for concern about the situation in those parts of sub-Saharan Africa where there are many poor families subsisting on monotonous diets based mainly on tryptophan-poor varieties of maize – which can easily lead to simultaneous niacin and tryptophan deficiency (551).

It should be noted that supplementation with niacin has been reported to have a similar protective effect as taurine supplementation against lower airway fibrosis induced by the ROS-generating anticancer drug bleomycin (63, 64, 66, 68–72, 74–76), suggesting that high doses of niacin might have a little-noticed antioxidant protective effect. Since bleomycin has a chemical mechanism of action overlapping strongly with that of ionizing radiation because it functions in combination with iron as a generator of reactive oxygen species (ROS) which next damage the DNA molecules (866–868), these observations suggest that high doses of niacin possibly also might have a similar protective effect against the fibrogenic and perhaps also other harmful effects of high doses of ionizing radiation. This is supported by the epidemiological observation that high dietary intake of niacin is associated with decreased chromosome translocation frequency in airline pilots (869).

Deficiencies of folate and vitamin B_12_ can mimic the effect of ionizing radiation in damaging DNA by causing single- and double-strand breaks, oxidative lesions, or both (870), but the same can also happen because of deficiencies of vitamin B_6_, niacin, vitamin C, vitamin E, niacin, iron or zinc (870). It was estimated in an article by Bruce Ames published in 1999 that the percentage of the population of the United States that had a low intake (< 50% of the RDA) for each of these eight micronutrients then ranged from 2% to more than 20% (870). A level of folate deficiency causing chromosome breaks occurred in approximately 10% of the population of the United States, and in a much higher percentage of the poor (870). His estimate was that such common micronutrient deficiencies that are likely to damage DNA by the same mechanism as for radiation and many chemicals appear to be orders of magnitude more important than the latter (870). However, the problem of dietary deficiencies mimicking the DNA-damaging effects of ionizing radiation must be expected to be even worse in many of the poor countries in the world than it is in the United States.

*In vitro* experiments indicate that genomic instability in human cells is minimized when folic acid concentration in culture medium is >227 nmol/l (854). Intervention studies in humans have shown: (a) that DNA hypomethylation, chromosome breaks, uracil misincorporation and micronucleus formation are minimized when red cell folate concentration is >700 nmol/l; and (b) micronucleus formation is minimized when plasma concentration of vitamin B_12_ is >300 pmol/l and plasma homocysteine is <7.5 micromol/l (854). These concentrations are achievable at intake levels in excess of current RDIs, *i.e*. more than 200-400 microgram folic acid per day and more than 2 microgram vitamin B_12_ per day (854). A placebo-controlled study with a dose-response suggests that based on the micronucleus index in lymphocytes, an RDI level of 700 microgram/day for folic acid and 7 microgram/day for vitamin B_12_ would be appropriate for genomic stability in young adults (854). It is thought that dietary intakes above the current RDI may be particularly important in those with extreme defects in the absorption and metabolism of these vitamins (854).

This must undoubtedly be highly relevant for the treatment of patients suffering from gastrointestinal damage after exposure to high doses of ionizing radiation. For minimizing the long-term mutagenic burden resulting from a given total absorbed dose of ionizing radiation, it would probably be useful to give workers who will be exposed to high doses of ionizing radiation an intramuscular injection with a large dose of methylcobalamin before exposure, or as soon as possible after they have been exposed. This can be safely done since cobalamin is very non-toxic, even to the extent that it can be safely administrated in huge doses as an antidote against cyanide poisoning.

It should be noted that fish protein concentrate type B is an excellent source of several of those nutrients that are needed for DNA synthesis and repair, but are often deficient in the diets of large groups of people both in sub-Saharan Africa and other poor countries (*e.g*. in the Caribbean), with the concentration of vitamin B_12_ being exceptionally high compared to other foods (550, 551). High-quality FPC can be produced very cheaply from cheap varieties of so-called industrial fish that comprise roughly 1/3 of the global wildfish catch, and that are now used for production of fish meal and oil that are mainly used as feed for animals (almost all of the fish meal and perhaps 70% or more of the fish oil). There are very strong reasons that this should be changed, if we want to optimize the health and minimize the population burden of unnatural mutations not only in the rich countries in the world, but also in the poor ones.

### Similar multifactorial strategies for suppression of harmful inflammation with minimal suppression of useful antiviral and antibacterial immunity can hopefully be used both for treatment of severe radiation injury, hypervirulent avian influenza and SARS

Following exposure to high doses of ionizing radiation, it will also be important to suppress the posttraumatic inflammatory overreaction that may be expected to come following severe radiation injury at the same time as immunological recovery and other tissue repair processes should be stimulated as well as possible, while an attempt should also be made to hinder fibrosis and scar tissue development as a consequence of the radiation injury.

There might be an apparent conflict between the need to suppress inflammation and the need to stimulate good immunological recovery, and anti-inflammatory preparates with too strong immunosuppressive effects, like glucocorticoid preparates, should therefore be avoided. An optimal diet may, however, be antiinflammatory *i.a*. because it may help to suppress prostaglandin overproduction and thus may help to reduce neurogenic inflammation (534), at the same time as it also facilitates immunological recovery. This is dramatically illustrated by the author's personal experience with the great value of the same type of food (*viz*. fish protein concentrate type B) both for treating severely ill patients suffering from the combination of malnutrition and infectious disease in Africa (663) and for treatment of rheumatoid arthritis in his mother, who was permanently cured for nearly 17 years until she died. It would perhaps be helpful as part of emergency preparedness efforts not only for encountering nuclear disasters, but also for reducing the number of deaths caused by pandemics with hypervirulent avian influenza or SARS, if countries all over the world (but especially those with nuclear power plants) could establish large stores with this product (FPC type B), packed under inert gas to prevent oxidative rancidification, and perhaps having it both in form of powder and granulate to make it practically easier to use it in patients.

Space does not allow any extensive discussion here of all those other substances, in addition to taurine, that might also be useful as part of multifactorial therapeutic intervention strategies following severe radiation injury either because they are radioprotective or for other reasons (*e.g*. because of anti-inflammatory or immune-stimulating effects). Attention may, however, be drawn to those multifactorial treatment strategies that have been proposed for hypervirulent avian influenza in earlier survey articles (520, 532), and also to a short article about “radiation biochemistry” written by the present author (817). For optimizing antioxidant and antinitrative protection in patients suffering from hypervirulent avian influenza, it was suggested in the articles about malignant influenza (520, 532) to use a combination of good diet and supplementation with high doses of taurine, high doses of the endogenously synthesized antioxidant coenzyme Q_10_ and high doses of the antioxidant and immunostimulatory hormone melatonin. A similar combination would probably be useful for patients who have been exposed to high levels of ionizing radiation as well.

The combination of optimal diet and high doses of melatonin (with strong radioprotective effects (711–771)) would be expected to helpful not only for optimizing radioprotection, but also for improving antibacterial and antiviral immunological functions (520, 532). Melatonin exercises its immunoregulatory effects at multiple levels both in the pineal gland, in the bone marrow and directly on peripheral leukocytes, with some of the effects being direct and others indirect because melatonin stimulates the synthesis of other signal substances with immunoregulatory functions, such as thymic hormones, opioid peptide cytokines produced by cells in the bone marrow, and other cytokines (520, 532, 533, 871–881). Melatonin does not only stimulate the production of natural killer cells, monocytes and other leukocytes, but alters the balance of T helper (Th)-1 and Th-2 cells mainly towards Th-1 responses and increases the production of relevant cytokines such as interleukin-2 (IL-2), IL-6, IL-12 and interferon-*gamma* (880).

Of special interest is the observation that high-dose melatonin supplementation protects against drug-induced leukopenia and thrombocytopenia in cancer patients who have been treated with cytotoxic drugs or interleukin-2, as well as in patients suffering from various other diseases (882–891). These effects are most plausibly explained by a combination of antioxidant protective effects and direct or indirect (via opioid cytokines?) stimulating effects of melatonin on the growth of myeloid precursor cells, possibly including macrophages (871, 872) giving rise to platelets and myeloid-derived leukocytes. The latter effect might be expected to be highly relevant also for patients who suffer from leukopenia and thrombocytopenia because they have been exposed to high levels of ionizing radiation (882–891). Another substance produced by the pineal gland, *viz*. 5-methoxytryptamine, has also been found to have a similar effect (900, 901). Protective effects against radiation-induced leukopenia and thrombocytopenia have, moreover, also been reported for the hydroxylated dehydroepiandrosterone metabolite androstenediol (892–895) and tocopherol succinate (897).

The combination of guanosine + inosine has also been found to be similarly protective (896). This observation is highly compatible with the hypothesis which has been discussed above that immunosuppression and intestinal injury following exposure to high levels of ionizing radiation may happen because of nucleotide depletion caused by a high rate of nucleotide consumption for DNA repair. If this explanation for the protective effect of the guanosine + inosine combination is correct, it would probably have been even better to give nucleoside precursors for all of the four different nucleotides that are used as building blocks during DNA synthesis and repair rather than for only two of them.

An important question concerns the safety of giving melatonin at high dosage levels to human patients (*e.g*. 20 to 40 mg/day for adult patients). Abundant animal experimental data suggest that melatonin given at high dosage levels might be useful in several different diseases as well as following severe trauma, but these animal experiments have in most cases not been adequately followed up by clinical studies with human patients, with the consequence that clinical experience with high-dose melatonin preparates is not very extensive, either as regards useful therapeutic effects or undesirable side effects. There are, however, some important exceptions to this, especially with the use of high-dose melatonin supplementation as adjunctive therapy for cancer, where there is a large number of studies from Lissoni and collaborators in Italy over a period of more than twenty years that involves a considerable cumulate number of patients that should be more than adequate for permitting an assessment of the frequency of adverse side effects compared to the frequency of positive ones (even though it may sometimes be difficult, when dealing with severely ill patients, to distinguish between adverse drug effects and adverse consequences of the disease itself). It would probably be useful if Lissoni himself could carry out some kind of metaanalysis of the results of his own studies, pooling the data for all those different patients who have been given high-dose melatonin over a period of more than twenty years not only concerning positive therapeutic effects, but also for putative adverse side effects, even when it is far from certain that there is any form of causal connection.

Some examples of publications from this group have been given here (882–891), but there are far more (far too numerous that they can be referenced here!) that easily can be found on PubMed by combining the search words melatonin and cancer with the author name. If there are important negative side effects of high-dose melatonin preparates, they would presumably be similar in patients suffering from other diseases as can be seen in cancer patients. But the first-glance overall impression from the clinical effects observed in a large total number of cancer patients is that the toxicity of this drug is very low (at least when compared to its positive therapeutic effects) and its safety correspondingly high, even when it is given at a dosage level of 20–40 mg/day to adult patients.

Another example of using melatonin at high dosage levels is a recent report from Spain about treatment of children suffering from Duchenne's muscular dystrophy with melatonin at at a dosage level of 70 mg/day (902). Ten patients aged 12.8 +/- 0.98 yr, were treated with melatonin (60 mg at 21:00 hr plus 10 mg at 09:00 hr) over 6 months, and plasma levels of lipid peroxidation (LPO), nitrites (NO_x_), interleukin-1*beta* (IL-1*beta*), IL-2, IL-6, tumor necrosis factor-*alpha*, interferon-*gamma*, and plasma markers of muscle injury, were determined at 3, 6 and 9 months of treatment (902). Healthy age- and sex-matched subjects were used as controls. The results show a significant increase in LPO, NO_x_, and cytokine levels in the blood plasma of Duchenne patients compared with controls (902). Melatonin administration reduced these values to control levels at 3 months of treatment, decreasing further 9 months later (902). In parallel, melatonin also reduced plasma levels of creatine kinase (50%), lactate dehydrogenase (28%), aspartate aminotransferase (28%), alanine aminotransferase (20%), and myoglobin (13%) (902). The authors conclude that the high-dose melatonin administration must have reduced significantly the hyperoxidative and inflammatory process in Duchenne patients, and that it must have reduced the muscle degenerative process (902).

The possibility of using other immunostimulating hormones in addition to melatonin (such as dehydroepiandrosterone or one of its more active hydroxylated metabolites, such as androstenediol (532), but also thymic hormones (533)), should, however, also be considered not only for treatment of hypervirulent avian influenza and other dangerous viral infections, but also for promoting the recovery of immunological functions in patients who have been exposed to high doses of ionizing radiation. There is no good reason why androstenediol should be much less effective not only for reducing the lethality of infection with very dangerous viruses (532, 533), but also as a radioprotectant (892–895) in human patients than has been found in animal experiments.

Similarly as earlier has been proposed for use as part of a multifactorial treatment strategy for malignant influenza (for stimulating the useful antiviral immune response without enhancing harmful inflammation) (532), it is possible that *beta*-glucans (which in this case probably would need to be orally administrated) might be valuable for stimulating recovery of the immune systems in patients who have been exposed to high doses of ionizing radiation. It is, furthermore, possible that some of those Toll-like receptor agonist preparates for stimulation of Th1 (antiviral and antitumour) immune responses that have been used for treatment of cancer patients, such as OK-432 (903, 904) also might be useful here, provided that they can be applied at a dosage level and by a route that does not lead to enhancement of harmful inflammation more than it enhances the capacity of the immune system to fight harmful bacteria and viruses.

Not only for improving antioxidant protection (520), but also for reducing the intensity of non-specific, but harmful inflammatory responses (532), it was proposed in the influenza articles to use a combination of good diet with high doses of taurine. There might be reason for hope that all these interventions (that should presumably be combined with each other for obtaining an optimal overall therapeutic response) also might be helpful for patients suffering from severe radiation injury.

### Possible relevance of the protein kinase CK2-mediated growth-stimulatory effects of polyamines for repair of intestinal mucosal damage in malnourished patients and patients suffering from radiation injury

Not mentioned in the influenza articles, but in my article about radiation biochemistry (817), polyamines are also potent radioprotective agents with polyamine depletion being correspondingly harmful for cells that are exposed to ionizing radiation, with the radioprotective effect apparently being due to a combination of different mechanisms including hydroxyl radical scavenging and structural effects (both as a direct consequence of polyamine binding to the DNA molecule and conformation changes in the DNA molecule itself) that reduce the access of reactive molecules to vulnerable sites in the DNA molecule (905–919). It may be relevant that polyamines, especially spermine, have been found to be highly potent antioxidants also capable of scavenging singlet oxygen (920–939); however, they can sometimes also have prooxidant effects (940).

Pharmacological inhibition of polyamine synthesis in the tumour cells must be expected to lead to enhanced sensitivity to radiation therapy (817, 905–907, 909), but polyamine depletion will also enhance the radiation sensitivity of normal tissues adjacent to the tumour. It is, however, very common (especially in fast-growing tumour cell populations) that polyamine synthesis rates are higher in tumour cells than in normal cells, for which reason pharmacological inhibition of their synthesis might be expected to be differentially more harmful to the tumour cells than to the adjacent normal tissues, thus hopefully offering an opportunity to improve the therapeutic ratio for the radiation therapy (by enhancing damage to the tumour cells more than to adjacent normal tissues). If the uptake capacity for polyamines in the tumour cells is not too high, it should in principle be possible to use a combination of pharmacological polyamine synthesis inhibition and polyamine supplementation for reducing the polyamine concentration in the tumour cells while maintaining polyamine concentrations in normal cells at a normal level (817). Some other methods for hopefully improving the therapeutic ratio of radiation therapy (after the expression of superoxide dismutase and NO synthase in the tumour cells has been measured) have also been mentioned in the same article (817).

Polyamines are essential to cellular growth processes (941–943), which may partly be explainned by their structural role in chromosomes when forming complexes with DNA. It was previously believed that polyamines are synthesized by every cell in the body when required, but it has later been shown that, as in the case of non-essential as well as essential amino acids (and in the case of other conditionally essential nutrients, such as nucleotides and taurine), the diet can supply sufficient amounts of polyamines to support cell renewal and growth (941). The relative contribution of dietary compared to endogenously produced polyamines would be expected to be especially high in the intestinal mucosa, where the epithelial cells also have especially fast turnover and the growth rate for the progenitors of mature epithelial cells must be correspondingly high. The concentration of polyamines in human milk is also relatively high (944–949), about 10 times higher than in infant formula (946), suggesting a role of polyamines in the normal development of the intestine (and associated microflora?), and perhaps also in the normal development of the immune system in the infant.

It is therefore possible that a diet rich in polyamines or polyamine supplementation might be helpful for patients suffering from severe radiation injury, especially for stimulating enterocyte precursor growth and hence tissue repair processses in the intestinal mucosa. The importance of polyamines for the control of growth and repair processes in the intestinal mucosa as well as of the functional properties (*e.g*. disaccharidase expression) of the intestinal brush border is now well documented by experimental studies (950–969). It may be worth mentioning-as an example possibly illustrating the practical clinical relevance of these experimental observations-that it was a diet not only rich in taurine, but also in DNA, RNA and polyamines (from capelin that had been caught during the spawning season) that I was using myself when treating a large group of patients suffering from the combination of malnutrition, measles and diarrhoea while working for the Red Cross in Africa in 1974, with dramatically positive therapeutic results as earlier explained.

An important part of the mechanism, in addition to their role as DNA-binding structural components in chromosomes, for explaining the stimulating effect of polyamines on cellular growth processes must be their role as intracellular signal molecules (or “second messengers”) that regulate various enzymes with regulatory functions, such as protein kinase CK2 (which was earlier called casein kinase-2) (970, 971). Protein kinase CK2 regulates hundreds of proteins both in the nucleus, in the cytosol and in the plasma membrane, thus being involved in the regulation both of gene transcription, RNA processing and protein synthesis by the ribosomes. It plays a global role in activities related to cell growth, cell death, and cell survival – to an extent giving it the status of a "master regulator" in the cell (972). As examples of proteins associating with and/or regulated by protein kinase CK2 may be mentioned the nucleolar proteins nucleolin (973, 974), Nopp140 (975) and nucleolar protein B23 (976), topoisomerase I (977), insulin receptor substrate 1 (IRS-1) (974), the ATP-binding cassette protein ABC50 (978), which interacts with eIF2 (eukaryotic initiation factor 2), a protein that plays a key role in translation initiation and in its control, and in regulation of ribosomes (978), the von Hippel-Landau protein (979), which can be mutated in cancer patients, and the transcription factor NF-*kappa*B (972).

It is therefore not implausible that an important part of the explanation for the exceptionally rapid recovery of my malnourished patients with measles and diarrhoea in Tillia in 1974 could have been strong polyamine-mediated activation of protein kinase CK2 in intestinal mucosal epithelial cells and leukocytes, which may have led to enhancement of the activities of several different proteins associated with cellular growth processes and therefore to more rapid synthesis of mRNA and protein combined with more rapid cell replication, as long as there was no deficiency of any nutrient that could have led to growth limitation either among the essential ones or among those conditionally essential nutrients that mucosal epithelial cells and leukocytes need for rapid growth. It would obviously be therapeutically relevant, if it might be possible to achieve the same also in patients with severe gastrointestinal symptoms because of radiation injury.

The "master regulatory" (972) functions of protein kinase CK2 as a regulatory enzyme simultaneously regulating several proteins in the nucleus, in the ribosomes, in the cytoskeleton and in the plasma membrane makes it an excellent candidate to function at a very high level – as one of the “top generals” – in the hierarchy of proteins that regulate the protein synthesis-dependent anatomical changes (with strengthening of the synaptic contacts between some cell pairs and weakening of the synaptic contacts between other cell pairs) that are needed for establishment of long-term memory in the brain. It may thus be suspected that polyamines and protein kinase CK2 may be very important not only in the fast-growing intestinal mucosal cells, but also for learning processes in the brain.

Ornithine decarboxylase, which is the most important rate-limiting enzyme in the polyamine biosynthetic pathway, is expressed abundantly in the brain, but most of it is present in form of an inactive complex with the inhibitor protein antizyme (980), which may be regarded as an inactive precursor storage form of the active enzyme similarly as when NF-*kappa*B is rendered inactive by complex formation with I*kappa*B. The ratio of the abundance of inactive precursor to active ornithine decarboxylase in the brain might possibly be as high as about 50/1 (980), suggesting that the local rate of polyamine synthesis in parts of the nerve cells can be enhanced very fast following appropriate local stimulation. Since the inactive antizyme/ornithine decarboxylase complex can be dissociated by GTP, leading to activation of the enzyme (981), activation of nucleoside diphosphate kinases in the synaptic region might presumably be of crucial importance for initiation of this process. The nucleoside diphosphate kinases make GTP from GDP at the same time as ATP is degraded. The same mechanism for activating ornithine decarboxylase has been found also in *Escherichia coli* (982), and might presumably be phylogenetically extremely ancient.

## Conclusions

It is reasonable to assume that the radioprotectant action of taurine, when given before irradiation (or a weakening of this protective effect in animals or human patients suffering from taurine depletion before the exposure to ionizing radiation), partly may be explained by its function as an important intracellular antioxidant in mammalian nucleated cells and platelets. Evidence for this antioxidant effect of taurine is mostly indirect and relies on the demonstration of a pronounced antidote effect of high doses of taurine against a large range of toxic substances that taken as a group have little in common except that the substance itself or some of its metabolites function as prooxidants in one way or another, or inhibit important antioxidative enzymes.

The mechanisms responsible for the *in vivo* antioxidant effects of taurine are, nevertheless, poorly understood because taurine is not particularly effective as a scavenger of free radicals or other oxidants, except hypohalite ions and aldehydes. It is therefore proposed here that it may also function by an entirely different mechanism by participating in formation of mixed complexes with iron, especially in form of complexes where the iron atom is coordinated with three oxygen atoms in phosphate groups on one side and three oxygen atoms in the sulfonic acid group of taurine on the other side, thus shielding the iron atom completely from contact with molecular oxygen, ROS, organic hydroperoxide groups or reducing organic molecules until the complex has dissociated. Experimental confirmation of this hypothesis is, nevertheless, still lacking, and the stability constants for mixed complexes of this type are also unknown. If this hypothesis is valid and applicable also for iron complex-bound with DNA molecules, it might presumably help to explain why the taurine concentration in mammalian cells (at least some of them) is especially high in the mitochondria.

Severe radiation injury must be expected to lead to a severe post-traumatic inflammatory response, similarly as happens after other forms of severe trauma (*e.g*. severe burns and brain stroke), and it is known from these other forms of trauma that a strong post-traumatic inflammatory response both can be associated with significantly enhanced death risk (*e.g*. following severe burns) and exacerbation of permanent tissue damage following the injury (which is perhaps most typically seen following brain stroke and severe mechanical trauma to the brain). Thus, it is a reasonable working hypothesis that both the death risk and extent of permanent tissue injury following severe radiation injury may be reduced by good anti-inflammatory therapy following the exposure. But since immunosuppression and damage to mucosal organs where pathogenic organisms easily can enter also are among the common consequences of severe radiation injury, the challenge to the physician is to dampen unspecific and harmful inflammatory over-response without simultaneously inhibiting too much the capacity of the immune system for good antibacterial and antiviral immune responses. Ideally, the immune system ought to be strengthened at the same time as the harmful inflammatory reactions can be inhibited as much as possible. This double challenge is very similar to the challenge also facing the doctor when trying to hinder that a patient infected with hypervirulent H5N1 avian influenza or SARS shall die from the disease, and there may be good reason for hope that a multifactorial strategy that is helpful for achieving this double objective in patients suffering from hypervirulent influenza or SARS also may be helpful for patients who have been exposed to high levels of ionizing radiation and vice versa.

Taurine may be expected to exert significant anti-inflammatory and also analgesic effects by a combination of several different mechanisms: 1) reducing prostaglandin synthesis and PKC oxidative activation because of its antioxidant effects and therefore helping to reduce C-fibre-mediated neurogenic inflammation, 2) functioning as an agonist ligand of inhibitory glycine receptors on neutrophil granulocytes and macrophages (and perhaps on eosinophils and mast cells as well?), 3) functioning as an agonist ligand of inhibitory receptors on C-fibres and thus reducing neurogenic inflammation, 4) reacting with hypohalite ions to form taurine chloramine and taurine bromamine, which inhibit activation of the transcripton factor NF-*kappa*B and therefore may help to reduce the expression of a large number of proinflammatory proteins, 5) regulating the rate of membrane transport of calcium in ways that would be expected to contribute to reduction of the secretion of histamine and other proinflammatory substances from storage granula in leukocytes and also reduce the rate of leukotriene synthesis.

The anti-inflammatory and analgesic effects of taurine may be expected to be therapeutically useful in several different diseases both when harmful inflammation develops as a consequence of infection (as in hypervirulent influenza or SARS, but most likely also in various diarrhoeal diseases) and when it has a non-infectious cause, as in rheumatoid arthritis, in asthma and other allergic diseases and after ischemia (*e.g*. myocardial infarction), and also following severe trauma (both severe burn injuries and severe mechanical truma) for reducing the risk that the patient shall die from shock or multiorgan failure. They may also be expected to be relevant in various common pain conditions, especially when the pain develops a consequence of ischemia, *e.g*. because of abnormal static loads or spasms in skeletal muscle (and perhaps also during childbirth), in which case the antiischemic protective effect of taurine also may be relevant.

In allergic diseases, the rate of production of taurine bromamine must be expected to depend on the concentration of bromide in blood plasma, which is most likely often strongly reduced in humans compared to the natural level found in wild animals because of high consumption in the human population of table salt with a Br/Cl ratio much lower than in seawater. If the plasma bromide concentrations of asthma patients could be normalized to the same level that is found in wild animals, taurine therapy would be expected to have considerably better effect than if bromide deficiency is not corrected.

For rheumatoid arthritis, a case history is discussed for illustrating the possible clinical relevance of the anti-inflammatory effects of taurine. A hypothesis for explaining major features in the pathogenesis of rheumatoid arthritis that represents a more elaborate version of one first proposed by Hajizadeh et al. (552) is presented as background for discussion of possible mechanisms explaining why the patient was permanently cured after she had started to take high daily doses of fish powder as a source of selenium – but the product is also a good source of taurine, glycine and other nutrients with anti-inflammatory effects. It is proposed that a vicious circle develops where a combination of hypoxia and much NO and proinflammatory cytokine production leads to necrotic cell death, while proinflammatory substances released when cells die in necrosis in combination with neurogenic inflammation cause enhancement of the production of NO and proinflammatory cytokines, such as TNF-*alpha*, which leads to enhancement of the number of cells dying through necrosis and therefore more inflammation. Taurine may, however, help to break this vicious circle both because of the direct inhibitory effects of taurine itself and taurine chloramine on neutrophils and macrophages and because of C-fibre inhibition.

Taurine is reported to function sometimes as a factor enhancing the rate of cell growth processes, especially in leukocytes and nerve cells, and sometimes as a growth-inhibitory factor. These opposite effects are probably due to very different mechanisms, with the growth inhibitory effects of taurine being a consequence of its interference with important intracellular signal systems regulating cell growth, while the growth-stimulating effects of taurine might be a consequence of its role as a nutrient because of its function as one of the major intracellular osmolytes and because of the mechanisms connecting cell volume regulation directly with the regulation of the rates of protein synthesis and protein degradation-with enhancement of the volume of the cell leading to enhanced protein synthesis and reduced protein degradation, while shrinkage of the cell has opposite effects. Considering the opposite regulatory effects of taurine on the growth of different cell types, there is good reason to hope that taurine supplementation in a patient that has become taurine-depleted because of radiation injury will help the immune system to faster recovery at the same time as it may help to hinder the development of fibrosis (which would be similar to the well-documented anti-fibrotic effect of taurine in the lower airways of animals exposed to the anticancer drug bleomycin).

Taurine should not be regarded as a “magic bullet” that can be used alone for prevention or treatment of severe radiation injury. Rather, it should be used as one among several components in multifactorial prophylactic and therapeutic interventions with multiple aims, *viz*. (1) optimizing cellular antioxidant defense, (2) optimizing DNA repair during and following the exposure to high doses of ionizing radiation, (3) minimizing the post-traumatic inflammatory reaction, (4) optimizing the recovery of immunological functions, (5) optimizing all other forms of tissue repair and regeneration, especially in the gastrointestinal tract (if at all feasible), and (6) preventing the development of fibrosis and scar formation.

Something similar may also be expected to be the case in several other disease conditions where taurine supplementation might be therapeutically useful, but where the best therapeutic effect probably can be obtained not by using taurine alone (not even when given at a very high daily dosage level), but by using it as part of a multifactorial treatment strategy, *e.g*. in combination with other nutrients also having antiinflammatory, antioxidative or antiischemic protective effects.

A practical problem associated with the use of taurine as an anti-inflammatory or analgesic agent is its rapid urinary excretion when applied in large doses. It would probably be better to use slow-release formulations rather than such rapidly absorbed taurine preparates that are now available in the market. Since it is well-known by the pharmaceutical industry how slow-release drug formulations can be made, it should not be practically infeasible (or much expensive) to produce in a very short period of time (if ordered by Japanese health authorities) as large quantities as might be needed for such preparates in the current emergency situation.

For improving emergency preparedness against future nuclear accidents in Japan or other countries, it will nevertheless be important to test the clinical effect of such preparates as well as possible under more normal circumstances, *i.e*. in non-emergency situations.

Something similar can be said also about melatonin, which has a fast turnover not because of renal excretion, but because of enzymatic degradation related to its function as a hormone helping to control diurnal biological rhythms (for which reason the blood concentration shall rapidly go down in the morning when light comes and people wake up). High-dose slow-release formulations should probably be preferred also here, but they need to be tested in non-emergency situations for verification that they indeed have the sought-for protective effects.

If such slow-release, high-dose taurine and melatonin preparates are found to have the desired effect, stockpiles should be made all over the world not only for improving emergency preparedness for the next large nuclear power plant catastrophe, but also for pandemics with highly lethal viruses, *e.g*. hypervirulent avian influenza or a new form of SARS virus that is not only more infective than the previous one, but also more lethal (similarly as the wave 2 Spanish Flu virus was both more infective and more lethal than the wave 1 Spanish Flu virus).

For pandemic emergency preparedness, it is also important to have enough food stockpiled in order to make it practically feasible to impose drastic measures including house quarantine to minimize geographic dispersal of the dangerous virus (532, 983). Such food stores for improving disaster preparedness (which might well include fish protein concentrate type B) might, however, also be useful after severe nuclear disasters by securing an adequate supply of foods that have not been radioactively contaminated, and could thus serve a double purpose (at the same time as they would, of course, also be helpful for minimizing the number of deaths following severe weather catastrophes, as in the hypothetical case that Yellowstone should blow up with one of its 1 per 700 000 years frequency mega-eruptions).

It should probably be useful to stockpile fish protein concentrate B (FPC type B) as part of such emergency preparedness stores because of its special value both for immunonutrition and as part of multiple anti-inflammatory therapy, at the same time as it is a highly concentrated product that can be produced and transported cheaply. It should then be stored in rock shelters under nitrogen to prevent oxidative rancidification, under suitable humidity conditions and at low temperature to make it possible to store the product over very long periods of time without any form of quality degradation.

The chemical mechanisms causing cellular damage during exposure to ionizing radiation and during ischemia followed by reperfusion are in large measure the same. It is therefore not surprising that several substances have been found in animal experiments to be protective in both situations. But much of the knowledge that is now available from this from a very large number of studies with several different substances in many different organs and species is not practically utilised in clinical medicine. The health economic benefits for society if this could be changed are potentially enormous, notably for acute therapy of cerebral stroke and myocardial infarction.
